# Transcriptional Control of an Essential Ribozyme in *Drosophila* Reveals an Ancient Evolutionary Divide in Animals

**DOI:** 10.1371/journal.pgen.1004893

**Published:** 2015-01-08

**Authors:** Sathiya N. Manivannan, Lien B. Lai, Venkat Gopalan, Amanda Simcox

**Affiliations:** 1 Molecular Cellular Developmental Biology Program, Ohio State University, Columbus, Ohio, United States of America; 2 Department of Molecular Genetics, Ohio State University, Columbus, Ohio, United States of America; 3 Department of Chemistry and Biochemistry, Ohio State University, Columbus, Ohio, United States of America; 4 Center for RNA Biology, Ohio State University, Columbus, Ohio, United States of America; Fred Hutchinson Cancer Research Center, United States of America

## Abstract

Ribonuclease P (RNase P) is an essential enzyme required for 5′-maturation of tRNA. While an RNA-free, protein-based form of RNase P exists in eukaryotes, the ribonucleoprotein (RNP) form is found in all domains of life. The catalytic component of the RNP is an RNA known as RNase P RNA (RPR). Eukaryotic *RPR* genes are typically transcribed by RNA polymerase III (pol III). Here we showed that the *RPR* gene in *Drosophila*, which is annotated in the intron of a pol II-transcribed protein-coding gene, lacks signals for transcription by pol III. Using reporter gene constructs that include the RPR-coding intron from *Drosophila*, we found that the intron contains all the sequences necessary for production of mature RPR but is dependent on the promoter of the recipient gene for expression. We also demonstrated that the intron-coded RPR copurifies with RNase P and is required for its activity. Analysis of *RPR* genes in various animal genomes revealed a striking divide in the animal kingdom that separates insects and crustaceans into a single group in which *RPR* genes lack signals for independent transcription and are embedded in different protein-coding genes. Our findings provide evidence for a genetic event that occurred approximately 500 million years ago in the arthropod lineage, which switched the control of the transcription of *RPR* from pol III to pol II.

## Author Summary

The processing of the 5′ end of nascent tRNAs is catalyzed by ribonuclease P (RNase P), an essential enzyme. In the ribonucleoprotein form of this enzyme, the RNase P RNA (RPR) functions as a ribozyme aided by protein cofactors. All previously examined eukaryotic *RPR* genes are transcribed from their own promoters by RNA pol III. In contrast, the *Drosophila RPR* gene is embedded in an intron of a recipient gene. We have shown that the embedded sequence, the only copy of *RPR* in the genome, is transcribed by pol II from the promoter of its recipient gene and encodes the functional RPR. Analysis of other animal genomes revealed that an embedded *RPR* is also present in the genomes of other insects and crustaceans. This feature provides evidence that the mode of transcription of *RPR* changed as the result of insertion into a recipient gene approximately 500 million years ago. This new, inserted type of *RPR* must first have appeared in the arthropod lineage in a common ancestor of insects and crustaceans.

## Introduction

RNase P catalyzes the essential removal of the 5′ leader sequence from precursor tRNAs (pre-tRNAs) [1]–[5]. With the exception of some protein-only variants in eukaryotes [6], [7], RNase P is a ribonucleoprotein (RNP) complex that consists of a catalytic RNA (RNase P RNA, RPR) and as many as ten protein cofactors (RNase P proteins, RPPs) in eukaryotes, up to five protein cofactors in archaea, and just one in bacteria [1], [2]. Conserved sequences and structural elements (including the active site) in all RPRs are suggestive of a shared evolutionary ancestry. By contrast, homology among RPPs is restricted to those of archaea and eukaryotes.

Biochemical characterization of bacterial RNase P has provided insights into how a single protein cofactor aids RNA catalysis by enhancing affinity for metal ions and substrate recognition [8], [9]. Comparisons of bacterial RNase P to its multi-subunit archaeal and eukaryotic counterparts provide an opportunity to examine whether structural and functional attributes of the RPR have been appropriated by additional protein cofactors. Of additional interest is understanding the role of these RPPs in regulating the function of RNase P during development and in response to environmental cues. In our efforts to develop *Drosophila* RNase P as a multicellular eukaryotic experimental model, we examined the transcription of *RPR*, and our work has unexpectedly shed some light on the evolution of this ancient ribozyme.

Eukaryotic *RPRs* that have been analyzed to date, ranging from yeast to human, are transcribed by pol III [2], [10]–[13]. The *RPR* gene in all *Drosophila* species examined [14]–[16] has been annotated in the last intron of *ATPsynC*/*CG1746* [17], the pol II-transcribed gene that encodes subunit C of the F_0_ complex that is part of the mitochondrial ATP synthase [15]. We showed that the *RPR* locus within this gene does indeed produce, in a splicing-independent fashion, a functional RPR. In subsequent analysis of genomic databases, we found that such embedding of the *RPR* gene within a pol II-transcribed gene is also a characteristic in all insects and crustaceans examined. This common feature within a major group of arthropods suggests that the change from pol III to pol II transcription of *RPR* occurred approximately 500 million years ago [18].

## Results

### An intronic RPR is conserved and expressed in *Drosophila* species

**Figure 1:**
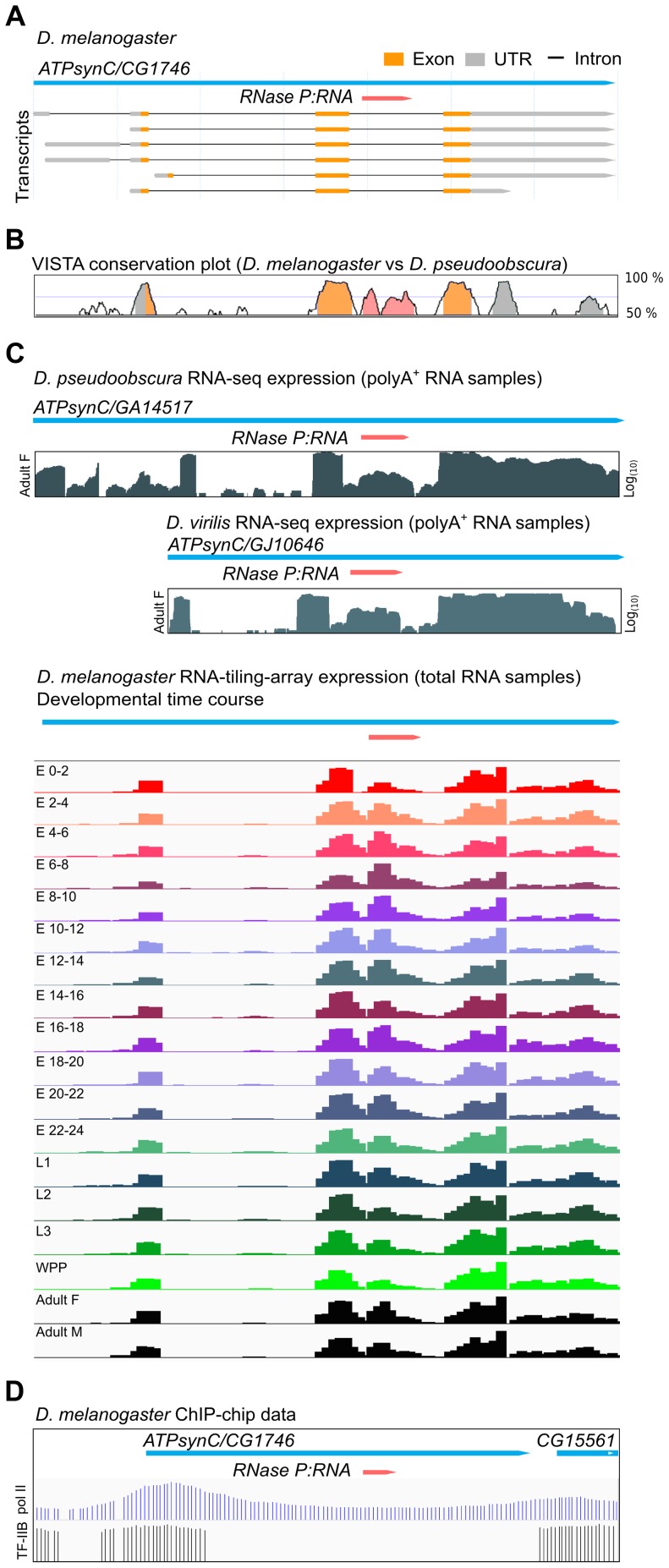
*Drosophila RPR* is embedded in an intron and ubiquitously expressed. A. The *RPR* gene (pink) in *D. melanogaster* is present in the last intron of *ATPsynC*/*CG1746*. B. This arrangement is conserved in *D. pseudoobscura* (and other members of the genus*).* The exons of *ATPsynC* (orange peaks) are highly conserved between these species, as is the region within the intron that contains *RPR* (pink). The preceding intron is not conserved. Untranslated regions of *ATPsynC* are shown in grey. Peaks showing 75% or greater conservation are colored. C. Analysis of polyA-selected RNA [52] from *D. pseudoobscura* and *D. virilis,* and of total RNA from different developmental stages of *D. melanogaster* show that the region corresponding to *RPR* is expressed at higher levels than the preceding intron [52], [53]. Presence of RPR in polyA^+^ RNA is likely due to carryover (see also Materials and Methods). D. ChIP on chip data (*D. melanogaster* embryos) showing binding sites of pol II [63] and transcription factor IIB (TF-IIB) [64] in the 5′ region of *ATPsynC/CG1746*. E, embryonic stage in hours after egg laying; L, larval instar; WPP, white pre-pupae; F, female; M, male.

Each of the twelve species of *Drosophila* for which genome sequence is available has a single copy of the *RPR* gene. In all cases, the *RPR* gene is inserted in the last intron of a recipient gene, *ATPsynC/CG1746,* with both genes arranged in the same 5′ to 3′ orientation (Fig. 1A). The *RPR* sequence is conserved, as are the *ATPsynC* exons and UTRs, but the other introns of the gene are not conserved (Fig. 1B). In keeping with a functional role, *RPR*-derived RNAs accumulate at higher levels (3- to 5-fold in polyA^+^ samples and 20-fold in total RNA samples) than those corresponding to the preceding intron (Fig. 1C). Like its recipient gene, *RPR* is expressed throughout development in *D. melanogaster* and in multiple tissues (Fig. 1C and S1 Fig.).

Although the expression data suggest that *RPR-*derived RNAs are expressed, we could not identify an *RPR* promoter by sequence analysis. The flanking sequences required for transcription by pol III, which are found in known eukaryotic *RPR* genes [10]–[12], [19], are absent in the vicinity of the *Drosophila ATPsynC-RPR* genes (S2A Fig.). The *Drosophila RPR* genes also lack internal pol III recognition sequences that are characteristic of tRNA genes (S2A Fig.) [16], [20]. Analysis of data from genome-wide chromatin immunoprecipitation (ChIP) assays in *D. melanogaster* shows binding of pol II in the 5′ region of the *ATPsynC-RPR* locus (Fig. 1D) [20], but ChIP studies mapping pol III binding in *Drosophila* do not identify a pol III target in the vicinity of the *RPR* genes [16], [20]. Together, these findings show that *Drosophila RPR* is expressed, but sequence analysis did not identify a pol III promoter that could drive its independent expression.

### *Drosophila RPR* expression requires a pol II promoter

**Figure 2:**
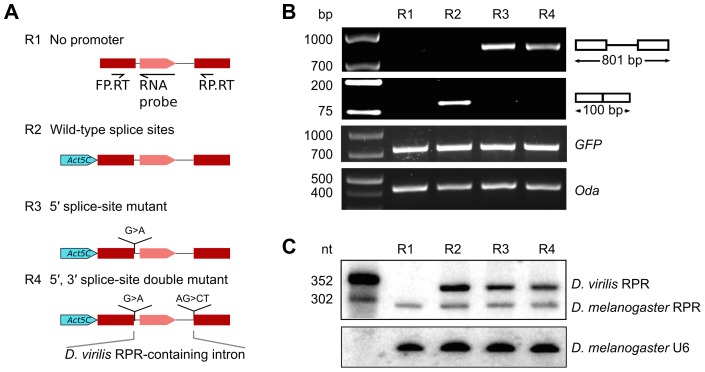
RPR is processed from a recipient intron. Various *RFP* reporter genes harboring the RPR*-*coding *D. virilis* intron were expressed in *D. melanogaster* S2 cultured cells to define sequences required for biogenesis of RPR. A. Schematic showing the reporter genes tested. B. *RFP* pre-mRNA and mRNA were analyzed by RT-PCR (using the primers FP.RT and RP.RT indicated in A). The presence of the protein was determined by fluorescent microscopy. C. RPR was detected by northern analysis. The antisense *D. virilis* RNA probe also detected the native *D. melanogaster* RPR because of sequence conservation. *Controls used*: GFP, expressed from a co-transfected plasmid to serve as a control for transfection efficiency; *Oda* (Ornithine decarboxylase antizyme) is a housekeeping transcript used to normalize input RNA for the RT-PCR experiment; U6 RNA was used as loading control the northern analysis.

The insertion of the *RPR* gene, which apparently lacks an independent promoter, into the last intron of *ATPsynC* in *Drosophila* suggests that *RPR* may be transcribed from the recipient gene promoter. To test this idea experimentally, we generated a reporter gene with the RPR-coding intron from *D. virilis* inserted between two red fluorescent protein (RFP) exons (Fig. 2A). The reporter gene was tested in *D. melanogaster* S2 cells in which *D. virilis* RPR can be distinguished from the endogenous *D. melanogaster* RPR by size and sequence differences. Transfected S2 cells expressed RFP from the reporter gene when driven by an *Actin 5C (Act5C)* promoter (pol II) [21]. RFP expression indicated that all the *cis*-elements required for correct splicing of the intron were present in the construct.

We analyzed RNA products from the reporter gene using RT-PCR and northern analysis. As expected from RFP expression, the mature *RFP* mRNA was expressed (R2 in Fig. 2B). *D. virilis* RPR was also expressed, demonstrating that the intron contained all the sequences necessary for production of mature RPR (R2 in Fig. 2C). The *D. virilis* RPR co-purified with RNase P activity (S3 Fig.), indicating that it assembled with endogenous RPPs to form a functional holoenzyme. *D. virilis* RPR was also expressed from a reporter gene with a *UAS-Hsp70* promoter [22], showing that the production of RPR is not dependent on the identity of the pol II promoter (S4 Fig.). In a reporter gene lacking a promoter sequence (Fig. 2A), no RPR was detected by northern analysis (R1 in Fig. 2C). This finding ruled out the possibility that the *RPR* gene was transcribed by a cryptic promoter in the intron that we could not identify by sequence analysis. Importantly, the failure to produce RPR also showed that transcription of *RPR* was dependent on the pol II promoter of the recipient gene.

### Splicing is not required for accumulation of *RPR*

To assess if splicing is required for processing of RPR from the intron, we designed splicing-deficient reporter genes and analyzed the RNA products using RT-PCR and northern analysis. We tested two reporter genes, one with a 5′ splice-site mutation and another with both 5′ and 3′ splice-site mutations (R3 and R4 in Fig. 2A). These mutations effectively blocked splicing as only the pre-mRNA for *RFP* was detected in the cells and no RFP expression was observed (Fig. 2B). In contrast, mature RPR accumulated (Fig. 2C), indicating that splicing is not required to process RPR from the primary transcript.

### The embedded *Drosophila RPR* gene encodes the ribozyme required for RNase P activity

**Figure 3:**
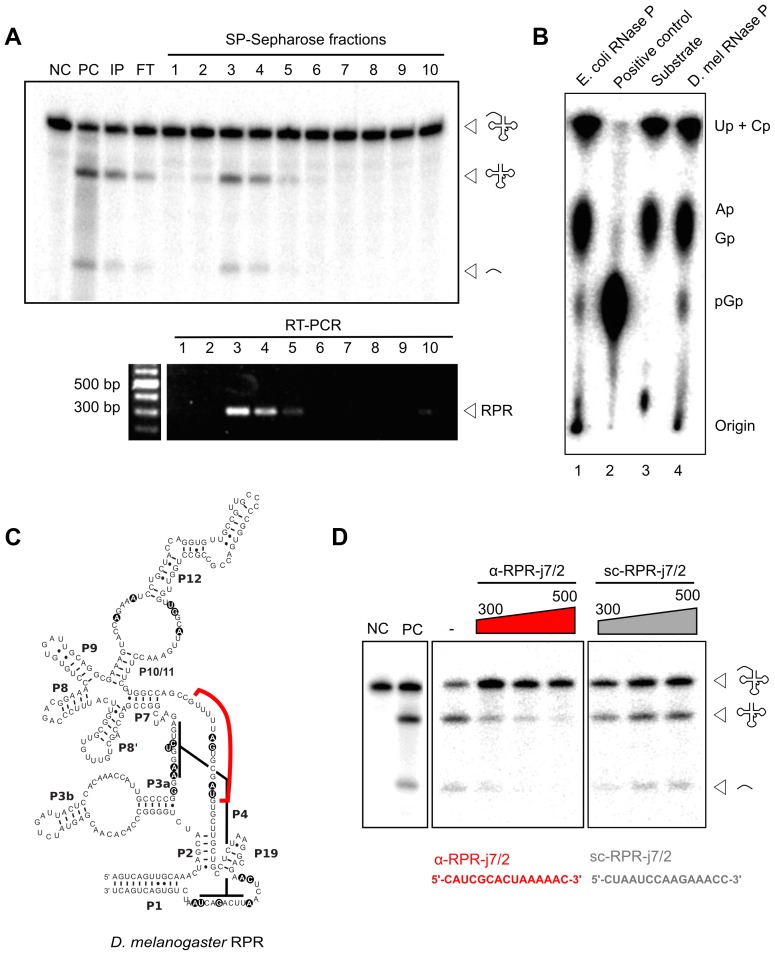
*D. melanogaster* RPR co-purifies with RNase P and is required for its activity. A. RNase P activity was partially purified from *D. melanogaster* S2 cells using sequential DEAE- and SP-Sepharose (above). Pre-tRNA processing assays established that the peak of activity eluted in 300–500 mM NaCl (fractions 3–5). RNA isolated from all fractions was subjected to RT-PCR using RPR-specific primers. Amplicons corresponding to the expected RPR size were detected in fractions 3–5 that showed maximal RNase P activity. B. Thin-layer chromatographic analysis of RNase T2-cleaved tRNA^Gly^ containing a 5′-pGp; the tRNA^Gly^ was first generated from cleavage of internally [α-^32^P]-GTP-labeled pre-tRNA^Gly^ by *in vitro* reconstituted *E. coli* RNase P or partially-purified *D. melanogaster* RNase P (lanes 1 and 4, respectively). The negative control (lane 3) shows RNase T2-cleaved internally labeled pre-tRNA^Gly^ that lacks a 5′-pGp, and the positive control (lane 2) shows RNase T2-cleaved 5′-labeled pre-tRNA^Gly^ that has a 5′-pGp. C. The predicted secondary structure of *D. melanogaster* RPR contains universally-conserved and functionally-important nucleotides (indicated by black circles). An antisense RNA oligonucleotide (red line; α-RPR-j7/2, complementary to a predicted single-stranded region between paired regions P7 and P2) was designed to inhibit RNase P activity. D. Partially-purified RNase P was inactivated with increasing concentrations of α-RPR-j7/2, but not with a scrambled oligo (sc-RPR-j7/2) that has the same nucleotide composition as α-RPR-j7/2. NC, negative control with no enzyme added; PC, positive control with *in vitro* reconstituted *E. coli* RNase P; IP, input; FT, flow-through.

The embedded *Drosophila RPR* is the only *RPR* copy in the genome suggesting that it fulfills the essential function as the ribozyme component of RNase P. We examined the association of the RPR with *Drosophila* RNase P to verify its functional role in the enzyme. The holoenzyme was partially purified from *D. melanogaster* S2 tissue-culture cells using sequential, ion-exchange chromatography on DEAE-Sepharose (anionic) followed by SP-Sepharose (cationic). The presence of RNase P activity in fractions from each matrix was detected using a pre-tRNA processing assay (Fig. 3A) [23]. Peak activity from both matrices was found in fractions eluted with 300 to 500 mM NaCl. *D. melanogaster* RNase P cleaved pre-tRNA^Gly^ to yield two products identical in size to those generated by the *Escherichia coli* enzyme, which was used as a reference standard (Fig. 3A). The mature tRNA resulting from cleavage by *D. melanogaster* RNase P had a 5′ phosphate on G_+1_, an end group expected from RNase P catalysis (Fig. 3B). This inference was based on finding guanosine-3′,5′-bisphosphate (pGp) in a thin-layer chromatogram of the products from RNase T2 digestion of *D. melanogaster* RNase P-generated mature tRNA^Gly^.

RPR present in the SP-Sepharose fractions was then detected using reverse-transcription and PCR (RT-PCR). The enrichment of RPR in fractions that also showed RNase P activity is consistent with its co-purification with the holoenzyme (Fig. 3A). To test if this co-purified RPR is required for RNase P activity, we designed an antisense RNA oligonucleotide (α-RPR-j7/2) that is complementary to a predicted single-stranded region that is part of the RPR active site (Fig. 3C). Incubation with α-RPR-j7/2 inhibited RNase P activity in a concentration-dependent fashion (Fig. 3D). In contrast, another oligonucleotide with the same nucleotide composition as α-RPR-j7/2 but a scrambled sequence (sc-RPR-j7/2) was ineffective at inhibiting activity even at the highest concentration tested. Together, these results confirm that the intronic *RPR* encodes the RNA component of *D. melanogaster* RNase P and is required for its activity.

### *RPR* genes in insects and crustaceans lack signals for pol III transcription

**Figure 4:**
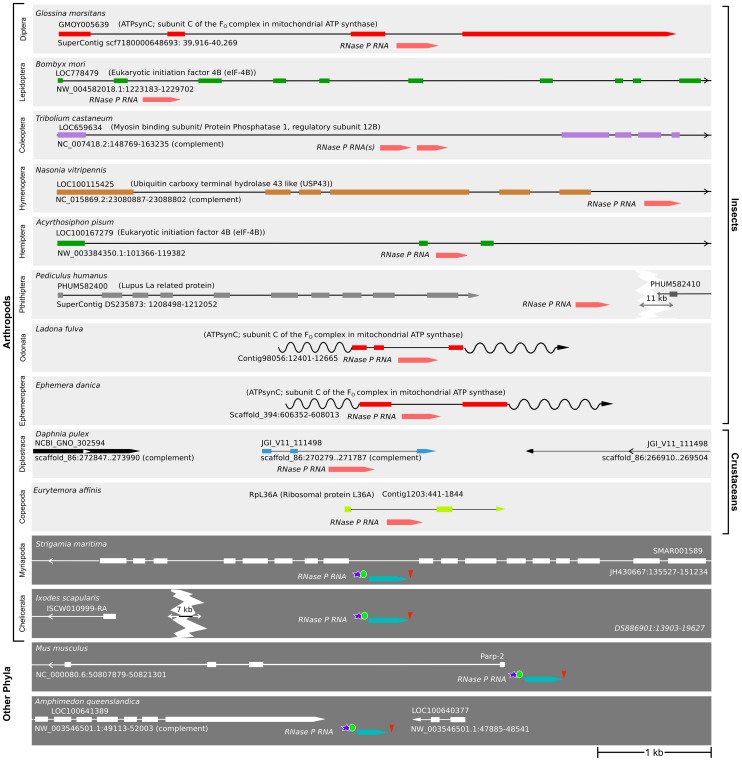
Insects and crustaceans have *RPR* genes embedded in *pol II* recipient genes, while other animals have independent *pol III* genes. The location of *RPR* and the neighboring genes in representative species of insects, crustaceans, and other animals are shown. In insects and crustaceans (light grey), *RPR* genes lack pol III signals and are in an intron (see also S2 Fig.). The *P. humanus* gene lacks pol III signals and is currently annotated between two genes. Each recipient gene is color-coded (as in Fig. 5; homologous genes have the same color). In other sub-phyla of Arthropoda (Myriapoda and Chelicerata) and other phyla (Deuterostomia and Porifera) (dark grey), *RPR* is an independent pol III-regulated gene (see also S2 Fig.). *RPRs* without pol III signals, pink; *RPRs* with pol III signals, blue; proximal sequence element (PSE), blue star; TATA box 21–27 nucleotides upstream of RPR, green oval; 3′ poly-T stretch of 4–5 nucleotides, red triangle. Wavy lines indicate regions where either poor sequence quality or weak homology prevents accurate prediction of the exons. Scale bar is 1 kb.

**Figure 5:**
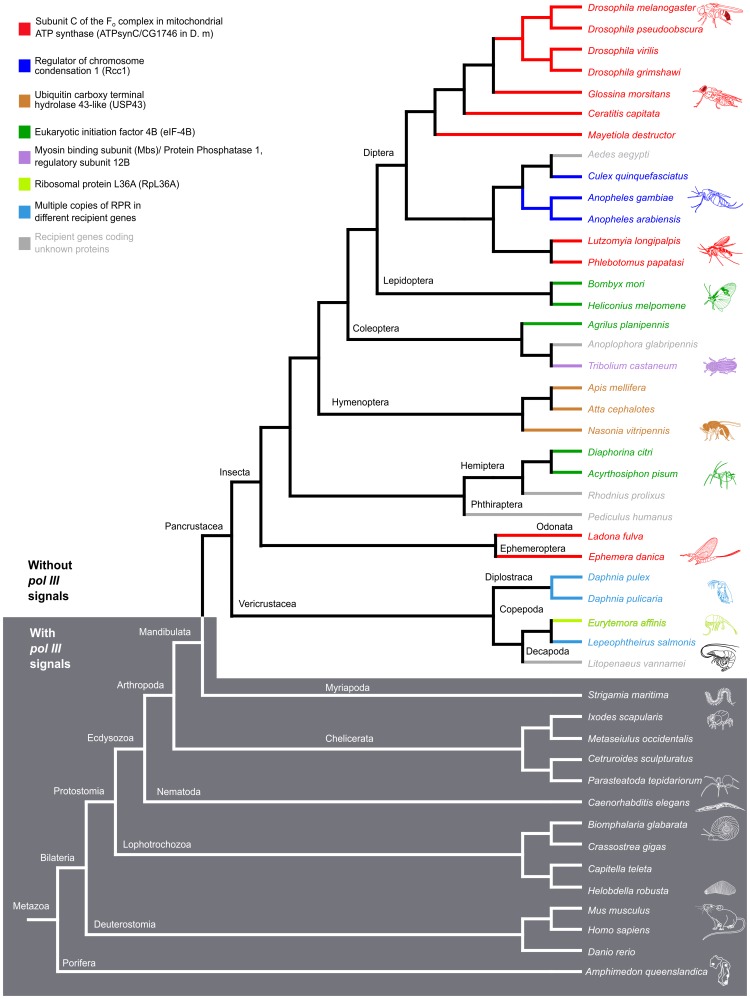
*RPR* genes lacking *pol III* signals are only present in the arthropod clade that includes insects and crustaceans. Phylogenetic relationship of animals showing two groups, those with *RPR* genes lacking pol III signals (light grey) and others with typical motifs found in type 3 pol III genes (dark grey). (See S2 Fig. for sequence motifs). The divide occurs in Arthropoda—species of Insecta and Vericrustacea (true crustaceans, including branchiopods and copepods) have *RPR* genes that lack pol III signals, whereas species of Myriapoda and Chelicerata have *RPR* genes with typical pol III signals. The *RPR* genes are associated with a variety of different recipient genes, indicated by different colored bars and named in the key (the same scheme is used in Fig. 4). In crustaceans (cyan), where there are multiple *RPR* genes in a single species, none is inserted in the ortholog of a gene identified as a recipient gene in insects.

To determine if the insertion of *RPR* in a recipient gene is unique to the *Drosophila* genus or more widespread in the animal kingdom, we analyzed *RPR* genes in the genomes of additional animals. All newly identified genes were verified to encode RPRs by their resemblance to typical eukaryotic RPRs in secondary structures and location of conserved nucleotides, including those essential for catalysis (S5 Fig.) [13]. Strikingly, we found a divide that classifies animals into two groups—(i) insects and crustaceans that have embedded *RPR* genes lacking signals for pol III transcription (Fig. 4, Fig. 5, S2A Fig. and S6A Fig.), and (ii) other animals that have typical signals for pol III-dependent transcription (Fig. 4, Fig. 5, and S2B Fig.). We draw these conclusions from an examination of species in the four subphyla of extant arthropods [Hexapoda (Insecta and Entognatha), Crustacea, Myriapoda, Chelicerata] and some non-arthropods that had not been previously examined.

Within the Hexapoda and Crustacea, we examined species in eight orders of insects and three orders of crustaceans. All these *RPR* genes lack signals required for pol III transcription (S2A Fig.). In 26 out of 27 insect species, the *RPR* gene is present in an annotated pol II-dependent recipient gene and oriented in the same 5′ to 3′ direction (Fig. 4). The one exception is *Pediculus humanus* (human body louse) where *RPR* is in a poorly annotated region. Nevertheless, it is likely that the *P. humanus RPR* is part of a recipient gene because it lacks signals for pol III transcription. In the case of *Tribolium castaneum* (red flour beetle) and *Heliconius melpomene* (Postman butterfly), there are two copies of *RPR* in the same recipient gene (Fig. 4 and S7B Fig.). The two *RPR* copies are present in tandem within the same intron in *T. castaneum*, while they are present in two different introns of the same gene in *H. melpomene*. We were unable to examine species in Entognatha, the other Hexapod class, because there is no genomic sequence available. In the five crustaceans that we examined, there are two or more *RPR* genes in a given species and all lack signals for pol III transcription (Fig. 4 and S6 Fig.). For example, there are ten *RPR*-like genes in *Daphnia pulex*, which is consistent with the extensive gene duplications that have occurred in its genome (S6A Fig.) [24]. At least one *D. pulex RPR* gene is expressed [24] and may be a functional gene (Fig. 4). Finding an inserted type of *RPR* gene in insects and crustaceans is consistent with their close evolutionary relationship [25]–[28] (Fig. 5).

Within the Myriapoda and Chelicerata, we examined one myriapod (centipede) and four chelicerates (spider, tick, scorpion, and mite). All species have an *RPR* gene with typical signals for pol III-dependent transcription (S2B Fig.). The same was found for five non-arthropod animal species we examined [two molluscs (snail and oyster), two annelids (polychaete worm and leech), and a sponge] in which *RPR* had not been previously analyzed (S2B Fig.). *RPR* genes in all these non-insect and non-crustacean species are present in intergenic regions, except for the centipede *Strigamia maritima*, where the gene is found in an intron in the opposite orientation to the recipient gene. These genes have typical signals for pol III transcription (Fig. 4 and S2B Fig.). These arthropods (Myriapoda and Chelicerata) and all other animals examined to date have what has been considered a typical RPR that is transcribed by pol III (Fig. 4 and Fig. 5).

### *RPR* resides in various recipient genes indicating a dynamic evolutionary history

Although the initial insertion of *RPR* into a recipient gene in the arthropod lineage appears to have been a single event, *RPR* moved again multiple times after this event as shown by its association with several different recipient genes (Fig. 5). In the eight orders of insects we examined, five different recipient genes were identified (Fig. 5). *RPR* recipient genes were also different within an order; for example, *RPR* is present in *Regulator of chromosome condensation 1 (Rcc1)* in mosquitoes, but it is in *ATPsynC* in the other species of Diptera.

Using the recipient gene as an indicator, *ATPsynC* appears to be the oldest recipient gene for *RPR* in the insects, as it is the common recipient gene in species belonging to the most divergent orders—the highly derived Diptera and the basal Ephemeroptera and Odonata (Fig. 4 and Fig. 5). Moreover, in *D. melanogaster*, *Ephemera danica* (mayfly) and *Ladona fulva* (dragonfly), *RPR* resides in the same intron providing further support for *ATPsynC* being the original recipient site in insects (Fig. 4, Fig. 5, and S7A Fig.).

Another common recipient gene for *RPR* is *eukaryotic initiation factor 4B (eIF-4B)*. *RPR* is present in *eIF-4B* in seven species belonging to three orders—Lepidoptera (moths and butterflies), Coleoptera (beetles) and Hemiptera (true bugs, including aphids) (Fig. 4, Fig. 5, and S7B Fig.). In five of the seven species, the insertion of *RPR* is in the same intron in *eIF-4B*. Although there is no significant conservation of its sequence, the intron can be identified based on the conserved amino acid sequence of the flanking exons (S7B Fig.). Presence of the RPR in the same intron is consistent with a common ancestor for these orders, but this is not supported by a well-established insect phylogeny [25]. An alternative explanation is that these were independent events and examples of recipient-site convergence. This idea is supported by the case of the Asian citrus psyllid *(Diaphorina citri)* and the bull-headed dung beetle (*Onthophagus taurus*) where *RPR* is in different *eIF-4B* introns (S7B Fig.), reflecting independent insertions of *RPR* into *eIF-4B* likely due to a bias for this recipient gene.

In *D. melanogaster*, the homologs of recipient genes in other insects and crustaceans are all expressed throughout development and in multiple tissues, with *ATPsynC* being one of the most highly expressed genes (S1 Fig.). This observation supports the idea that the expression pattern and level of expression may constrain possible recipient genes, so that only those genes with ubiquitous and high expression are suitable sites for insertion of *RPR* (S1 Fig.). In *Tribolium castaneum,* there are two *RPR* genes embedded in tandem in the *myosin binding subunit/protein phosphatase 1 regulatory subunit 12B-like* gene *(Mbs/PPP1R12B)*. Although *Mbs* shows a low level of expression relative to the other recipient genes, the two copies of *RPR* may compensate for this (Fig. 4 and S1 Fig.). Analyzing more insect genomes and transcriptomes will provide information about genomic contexts suitable for functional insertion of *RPR* and may reveal common features of recipient sites.

### RNase MRP RNA, a sister RNA to RPR, is regulated by pol III

RNase MRP has roles in mitochondrial DNA replication, nucleolar rRNA processing, and mRNA turnover, and is present only in eukaryotes. It is an RNP that shares eight protein subunits with RNase P [29]. Furthermore, the RNA subunit of RNase MRP (MRP RNA) resembles RPR and appears to have derived from a common ancestor by a gene duplication event early in eukaryotic evolution [17], [30]. The two RNAs, albeit similar in secondary structure, have distinctive features that enable their unambiguous identification.

Given our unexpected findings of a transcriptional switch for the *RPR* in insects and crustaceans, we conducted a survey of *MRP RNA* genes in 26 insect species (in addition to *Drosophila* [14], [31]). These newly identified genes encode *bona fide* MRP RNAs, as judged by secondary structures and the location of various previously established signature motifs; for example, a five-nucleotide “GARAR” consensus in L8 (the terminal loop which caps the P8 helix; [17]) is present in all of them. In all 26 cases, we found signals for pol III transcription (S8 Fig.) [14], [31]. Therefore, *MRP RNA* genes, in contrast to *RPR* genes, appear to have maintained pol III regulation throughout the animal kingdom, including insects and crustaceans.

## Discussion

Eukaryotic *RPR* genes have been widely held as independent genes transcribed by RNA pol III. Contrary to this generalization, we found that crustaceans and insects have *RPR* genes that lack signals for pol III transcription and are embedded in a recipient gene. In *Drosophila*, we demonstrated that the embedded *RPR* is dependent on the pol II promoter of a recipient gene for expression and that the encoded RNA copurifies with and is required for RNase P activity. Our findings change the long-held view of *RPR* as a prototype pol III-dependent gene [12], [32], and have implications for the biogenesis and evolutionary genetics of *RPR*.

### The biogenesis of RPR

In *Drosophila* species, the *RPR* gene is embedded in the last intron of the *ATPsynC* gene. We found splicing was not required to produce mature RPR using an experimental reporter system. In the native context, RPR could either be generated from the spliced-out intron or from the primary transcript, with additional processing required to trim sequences beyond the mature RPR termini. Certain classes of micro RNAs (miRNAs) [33] and intron-derived small nucleolar RNAs (snoRNAs) [34], [35] also require processing to generate their mature 5′ and 3′ termini. The intronic miRNAs, which also do not require splicing when assessed using reporters [33], are processed to their mature lengths by Drosha and Pasha/DGCR8 [36]. It is unlikely these endonucleases trim *Drosophila* RPR, because their recognition sequences are absent in the regions flanking the mature RPR. In the case of intron-derived snoRNAs, examples of both splicing-dependent and splicing-independent processing are found, wherein nucleolytic trimming guides the maturation of the snoRNA termini following the assembly of snoRNP proteins [34], [35]. Like snoRNP proteins aiding the processing of the snoRNAs, RPPs could play a role in the maturation of the intronic RPR, but details of the assembly of the RPPs on the intronic RPR remain to be investigated. To further understand the biogenesis of the intronic RPR, it will be important to identify the nucleases that act on the RPR ends to produce the mature form. We presume these enzymes were already present in the founder animal for processing other non-coding RNAs, and were co-opted to generate the mature RPR from the recipient gene transcript. If so, identifying the enzymes acting on RPR may also provide general information on the biogenesis of some other non-coding RNAs.

It has been reported that some other non-coding RNAs show differences in their transcriptional control and are transcribed by pol II in some organisms and pol III in others [37], [38]. The significance, if any, for the different mechanisms is unclear. One of the possible effects of a change in the transcriptional control of *RPR* is altered RNA activity (for example, from differences in modification). Testing this idea using RPR produced *in vivo* by pol II or pol III in a pre-tRNA processing assay will provide a tractable experimental model for determining whether the transcriptional shift between pol II and pol III has functional consequences for a non-coding RNA.

### Evolutionary genetics of RPR

**Figure 6:**
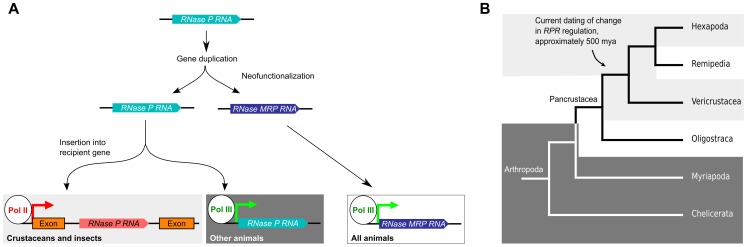
Model for the evolutionary history of *RPR*. A. An ancestral *RPR* gene is thought to have undergone gene duplication and one of the daughter genes assumed the new functions of MRP RNA (neofunctionalization). *MRP RNA* is transcribed by pol III in all animals, as is *RPR* in animals previously characterized. We found that the *RPR* gene in crustaceans and insects has undergone another genetic event that inserted it, devoid of pol III signals, into a pol II-transcribed gene. B. A cladogram showing arthropod evolution (based on, [26], [27], [65]). In Hexapods and true crustaceans (Vericrustacea) (light grey), *RPR* is embedded in a pol II-regulated gene. In contrast, in Myriapoda and Chelicerata, *RPR* is a pol III gene (dark grey). The *RPR* genes in Remipedia and Oligostraca have not been characterized due to lack of genomic sequences (unshaded). The arrow indicates a node that connects branches where *RPR* is found in a recipient gene. These groups are thought to have diverged 500 million years ago [18], [66]. We predict that Remipedia *RPR* is also embedded in a recipient gene similar to the sister group Hexapoda. An analysis of *RPR* in Oligostraca will enable us to determine if embedding of *RPR* occurred earlier in arthropod evolution in an ancestor of all pancrustaceans.

Based on sequence analysis, it has been hypothesized that *RPR* gene gave rise to the *MRP RNA* gene in eukaryotes, presumably through gene duplication followed by neofunctionalization of the new gene copy [17], [30] (Fig. 6A). While *MRP RNA* is under pol III regulation in all animals that we and others have examined, *RPR* has undergone a second genetic event that inserted it into a recipient gene in crustaceans and insects (Fig. 6A). Current data indicate that this genetic change, which caused embedding of *RPR* within the arthropod lineage, occurred approximately 500 million years ago in an ancestor of the insects and crustaceans, an estimate that is placed prior to the emergence of the insects at approximately 479 million years ago [18]. The species of crustaceans we examined are examples of the so-called true crustaceans (Vericrustacea) [26], which are closely related to the insects (Hexapoda); both are members of the epic Pancrustacea clade (Fig. 6B) [26]–[28]. The other major group of Pancrustaceans is the Oligostraca, that includes the seed shrimp, oar-feet, fish lice, and tongue worms, for which there is currently no genomic sequence. If Oligostraca species have an embedded *RPR*, this would support an earlier origin—in an ancestor of all pancrustaceans (Fig. 6B). As more genomes become available, we will be able to refine when a pol II-regulated *RPR* first occurred and test whether it was indeed a single event in arthropod evolution.

Generating an embedded *RPR* could have involved DNA- or RNA-mediated duplication and subsequent loss of any associated signals for pol III transcription [39]. It is also possible, given the catalytic function of RPR, that the insertion resulted from reverse splicing, similar to at least one route hypothesized for the spread of self-splicing group I and group II introns [40], although this activity has yet to be demonstrated for RPR. Regardless of the mechanism, the insertion caused a change in the regulation of *RPR* so that it became dependent on pol II transcription. This is not the case for *MRP RNA* (S8 Fig.), which shows the switch in transcriptional regulation occurred uniquely to RPR. As a first step towards determining any consequence of the change in *RPR* transcription, genome editing could be used to engineer, for example, *D. melanogaster* to have only a pol III-dependent *RPR* gene. Such a strategy would allow determination of the phenotypic consequences of reverting to the ancestral regulation of *RPR*.

Following the initial event that caused embedding in a recipient gene, *RPR* moved again multiple times into different recipient genes (Fig. 4, Fig. 5, and S7B Fig.). Insertion does not appear to be random because *RPR* inserted independently into the same gene more than once. In cases where *RPR* is present in two copies, such as *T. castaneum* (Fig. 4) and *H. melpomene* (S7B Fig.), both are present in the same recipient gene either in the same or two different introns, which is suggestive of local duplications. The crustaceans that we examined all had multiple *RPR* copies, however, these were associated with different recipient genes (S6A Fig.). While we have not identified a ‘signature’ of an insertion site, it appears that in all instances a pol II-regulated *RPR* has been retained and no case of a pol III-regulated *RPR* was found.

Our studies have shed some light on the evolution of RPR, a legacy of the RNA world and the first true trans-acting ribozyme discovered, and suggest that *RPR* transcription and subsequent processing entails the use of a different mechanism in a large group of animals. Although it is not known if this mode of biogenesis has functional consequences, our findings add to the variations in RNase P, an essential housekeeping enzyme, already noted for the diversity in its subunit composition [3], [5].

## Materials and Methods

### Cell culture and RNA isolation

*D. melanogaster* S2 cells [41] were grown in Schneider insect medium (Sigma) with 10% (v/v) fetal bovine serum. DNA transfections were performed using Effectene (Qiagen). Cells were harvested 30 h post transfection and total RNA was isolated using Trizol (Invitrogen).

### Construction of *RFP-RPR* reporter genes

The intronic region containing RPR was amplified from *D. virilis* genomic DNA using PCR and the following primers: forward primer virilis intron, 5′-CTGCTTCATCTACAAGGTTCGTATTGGTTACC-3′ and reverse primer virilis intron, 5′-CCGATGAACTTCACCTGTTGTATTGGTTGTC-3′. A DsRed ORF (from pP(RedH-Stinger) [42] was used as a template to generate two exons, separated at nucleotide 323, which creates a match to the consensus *Drosophila* splice junction [43]. The exons were generated using PCR and the following primer pairs: Exon I forward primer RFP, 5′-TCCGATATCATGGCCTCCTCC-3′ and Exon I reverse primer RFP, 5′-GGTAACCAATACGAACCTTGTAGATGAAGCAG-3′; Exon II forward primer RFP, 5′-GACAACCAATACAACAGGTGAAGTTCATCGG-3′ and Exon II reverse primer RFP, 5′ -ACCTCTAGACTACAGGAACAGGTGGTG -3′. The intron and the two exons were combined using overlapping PCR [44] and cloned into pPACPL, which contains the *Act5C* promoter [21]. DsRed exons were also generated with splice mutations. Splice mutations were created by site-directed mutagenesis with the following primer pairs: 5′ splice mutant forward, 5′-CTGCTTCATCTACAAGATTCGTATTGGTTACC-3′ and 5′ splice mutant reverse, 5′-GGTAACCAATACGAATCTTGTAGATGAAGCAG-3′; 3′ splice mutant forward, 5′-CAATGACAACCAATACAACCTGTGAAGTTCATCGGCGTGAACT-3′ and 3′ splice mutant reverse, 5′-AGTTCACGCCGATGAACTTCACAGGTTGTATTGGTTGTCATTG-3′. In addition, the fragment containing the DsRed exons with the *D. virilis* intron was cloned into pUAST [22] to generate a reporter gene under control of the *UAS* promoter. This reporter was expressed using a Gal4 gene (pGaTB) [22] cloned into the pPACPL vector using the following primers, Gal4 forward, 5′-TCCGATATCATGAAGCTACTGTCTTCTATC-3′; Gal4 reverse, 5′-AAATCTAGATTACTCTTTTTTTGGGTTTGGTGGGGTATCTTC-3′.

### Reverse transcription and PCR

cDNAs were prepared using an oligo dT primer (for mRNAs) or gene specific primers (for RPRs) by reverse transcription using an Omniscript RT kit (Qiagen). cDNAs were amplified with *Taq* DNA polymerase (NEB) using the recommended conditions and the following primer pairs: Forward DmelRPR, 5′-AGTCAGTTGCAAACTAGCATC-3′ and Reverse Dmel RPR, 5′- AGTCAGTCACAGATTAGTCTGAATTG-3′; Forward GFP, 5′-TAAGATATCATGGTGAGCAAGGG-3′ and Reverse GFP, 5′- ACCTCTAGATTACTTGTACAGCTCGTCC-3′; Forward Oda, 5′-GTCCTTCGGTAGAGCGACAT-3′ and Reverse Oda, 5′- GCACCATCTCGACTTCGTCT-3′.

### Northern blot analysis

*D. melanogaster* and *D. virilis* RPRs were detected using full-length anti-sense RNA probes labeled with [α-^32^P]-ATP in an *in vitro* transcription reaction. The DNA templates were generated from PCR-mediated amplification of the genomic DNA using the following primers (for both species): Forward primer-genomic, 5′-AGTCAGTTGCAAACTAGCATCTG-3′ and Reverse primer-genomic, 5′-TCACTATAGGAGTCAGTCACAGATTAGTCTG-3′. A T7 RNA polymerase promoter was introduced to the above PCR product using a second round of PCR using the same forward primer and the following reverse primer: 5′-GAGAATTCTAATACGACTCACTATAGGAGTCAGTCACAG-3′. *D. virilis* RPR was also detected using the following DNA oligo, 5′- CCGCGACACACAATCACCTCTCGGCTTTTGTATGTTGTTACAGCAAC-3′. U6 RNA was detected using the DNA oligo, 5′- GCAGGGGCCATGCTAATCTTCTCTGTATCG-3′. Both DNA oligos were 5′-labeled using [γ-^32^P]-ATP and T4 polynucleotide kinase. Eight micrograms of total RNA isolated from transfected cells was separated on a 7.5% (w/v) polyacrylamide gel containing 8 M urea, transferred to a nylon membrane (Hybond N+, GE Healthcare) and analyzed by northern hybridization. After pre-hybridization in the same hybridization buffer, RNA probes were hybridized in hybridization buffer (5X SSC, 1% (w/v) SDS, 5X Denhardt's solution, 200 µg/ml of sheared salmon sperm DNA) for 16 h at 65°C. DNA oligo probes were hybridized in QuikHyb buffer (GE Healthcare) for 16 h at 55°C. Membranes were washed with 2X SSC with 0.1% (w/v) SDS at 10°C below hybridization temperature. The binding of labeled probes to their complementary target RNAs was detected using phosphorimaging.

### RNase P purification

*D. melanogaster* S2 cells were collected by centrifugation and washed once with phosphate-buffered saline. Packed cells (100 µL) were lysed in 400 µL of lysis buffer [15 mM HEPES (pH 7.9), 3 mM MgCl_2_, 50 mM NaCl, 1 mM dithiothreitol, 0.2 mM phenylmethylsulfonyl fluoride, 0.1% (v/v) Tween-20, 10% (v/v) glycerol, 0.2 U/μL of Ribolock RNase Inhibitor (Thermo Scientific)]. Cells were homogenized using a type A glass Dounce homogenizer (Wheaton) on ice and debris was removed by centrifugation at 2,500 g for 10 min. The crude lysate was mixed with 100 µL of diethylaminoethyl (DEAE)-Sepharose resin (GE Healthcare), which had been pre-equilibrated with lysis buffer at 4°C for 30 min. The resin was collected by centrifugation (2,500 g for 5 min) and washed twice, each with 1 mL of lysis buffer to remove weakly bound constituents. Fractions were eluted stepwise with increasing NaCl concentration (from 50 mM to 1 M) in lysis buffer, and tested for RNase P activity (as described below). Fractions with detectable activity were pooled and dialyzed twice, each with 500 volumes of lysis buffer (without NaCl and RNase inhibitor) for 2 h at 4°C. The dialysate was then mixed with 100 µL sulfopropyl (SP)-Sepharose resin (GE Healthcare), washed with 1 ml of lysis buffer, and bound constituents eluted with increasing NaCl concentration (as described above for the DEAE-Sepharose purification).

### RNase P activity (pre-tRNA processing) assay and inhibition studies

Four μL of partially purified *Drosophila* RNase P fractions were assayed in a 20-μL reaction containing 10 mM HEPES (pH 7.9), 10 mM magnesium acetate, 200 mM ammonium acetate, 0.1% (v/v) Nonidet P-40 and 250 nM of *in vitro* transcribed pre-tRNA^Gly^ (tobacco chloroplast; without 3′-CCA), a trace amount of which had been internally labeled with [α-^32^P]-GTP (28). The reactions were incubated at 28°C for 10 min, and then terminated with 10 µL of 2X urea loading dye [8 M urea, 15 mM EDTA, 0.025% (w/v) xylene cyanol, 0.025% (w/v) bromophenol blue, 20% (v/v) phenol]. The products were separated on an 8% (w/v) polyacrylamide (19∶1) gel containing 8 M urea, and detected using phosphorimaging. Oligo-inhibition assays were performed as described earlier for bacterial and archaeal RNase P [23], [45], [46]. For these experiments, the RNA oligo (final concentration 300, 400 or 500 µM) was pre-incubated with 4 µL partially purified RNase P in assay buffer for 5 min at 28°C. After addition of substrate (pre-tRNA^Gly^) in assay buffer, the reaction was incubated for 15 min at 28°C, and then terminated and characterized as described above.

### Bioinformatics analysis

We performed sequence analysis using genomic data from multiple sources—i5K Pilot Project (Baylor College of Medicine, Human Genome Sequencing Center), NCBI Genome, Ensembl Genomes, VectorBase, Penaeus Genome Database (PAGE), BeeBase, wFleaBase, FlyBase, DOE Joint Genome Institute (Table S1), using the Infernal package (release 1.1) [47]. The secondary structures for newly discovered RPRs were drawn using a ClustalW2-aided multiple sequence alignment of the RNA sequences and Mfold [48]. The results of our search, which yielded 32 new *RPRs*, were independently validated by the fact that 9 of these 32 RPRs were identified using a different bioinformatics approach [49]. These putative RPRs were manually analyzed for size, the presence of conserved nucleotides (identity and location) and the length of P1 helix (which help define the 5′ and 3′ termini). The RPRs we identified diverged in the length and sequence from prototypes and were not identified using previous methods. Incorporating this information into covariance model-based searches will improve future RPR searches. Our results should also serve as a cautionary note for excluding putative *RPR* genes because they lack *pol III* promoters and terminators. MRP RNAs were identified and analyzed using an approach similar to that employed for RPRs.

The cladograms in Fig. 5 were generated using the NCBI taxonomy browser, Dendroscope [50], and current literature on arthropod phylogeny [25], [26]. The VISTA conservation graphs in Fig. 1B and S7A Fig. were generated using mVISTA [51]. For analysis of *RPR* expression during *D. melanogaster* development and in various tissues, data derived from the analysis of total RNA by tiling arrays were examined [52]. These data were obtained from the modENCODE consortium [53] as wig files and viewed using the Integrative Genomics Viewer [54] (S2 Table). For *D. virilis* and *D. pseudoobscura*, data from polyA-selected samples analyzed by RNA-seq were used (sam files). The SAMtools program was used to index and sort the RNA-seq reads [55] and the Integrative Genomics Viewer was used to visualize the reads. In both the total RNA and polyA-selected samples, reads corresponding to the *RPR*-containing intron are higher than those corresponding to the preceding intron in *ATPsynC.* The presence of RPR in the polyA^+^ sample may have resulted from incomplete removal of highly expressed RNAs, as has been observed in RNA-seq analyses for other non-polyA^+^ RNAs [56]. The reads for the polyA^+^ samples are as follows; *D. virilis*: *RPR,* 2435 reads/353 bp and preceding intron, 978 reads/683 bp; *D. pseudoobscura*, RPR 2478 reads/322bp and preceding intron 1980 reads/804 bp. For *T. castaneum*, RNA-seq data as raw reads (SRR1048514 and SRR1161702 in SRA format; refer to Table S1 for details) were downloaded from NCBI GEO, and ERR161589 as FASTA was downloaded from EMBL ENA. The SRR files were converted to FASTQ format using SRAtoolkit [57]. These reads were mapped to *T. castaneum* genome (Genome version Tcas3, from EMBL indexed using Bowtie2 [58]) and TopHat [59]. The mapped reads were then analyzed using Cufflinks [60] with annotated transcripts (Tcas3.22.gtf). The locus of *T. castaneum* recipient and housekeeping genes were identified in the version of the genome mentioned above, using TBLASTN (NCBI-BLAST+) (refer to Table S3 for loci) [61]. SAMtools was also used to extract genomic sequences flanking the identified RPRs and these sequences were aligned using ClustalW2 [62]. For each species, a consensus proximal sequence element (PSE) and TATA box was generated using an alignment of the U6 and 7SK RNA promoters. This consensus was used to search for candidate pol III promoter elements proximal to a given newly identified RPR sequence.

## Supporting Information

S1 Fig Expression of *RPR, ATPsynC/CG1746* and homologs of other *RPR*-recipient genes in *D. melanogaster* and *T. castaneum.* A. Expression of *ATPsynC/CG1746* and *RPR* in heads of 1-day-old virgin females and ovaries of 4-day-old mated females. Samples were total RNA analyzed for expression levels with tiling arrays [52]. B. Heat map showing expression of the indicated genes in *D. melanogaster* embryos, adults (male and female), S2 or Kc tissue culture cells, and various tissues (imaginal discs, central nervous system, digestive system and carcass of third instar larva (L3) as well as ovaries and testes of 4-day-old adults). *ATPsynC/CG1746* is expressed at high levels in *D. melanogaster*–the average expression level makes it one of the top 200 most highly expressed genes. Expression levels (reads per kilobase per million mapped reads, RPKM) for the *D. melanogaster* homologs of five *RPR-*recipient genes in other animals are shown: *RpL36A, Uch (Ubiquitin carboxyl-terminal hydrolase 1, USP43* in *N. vitripennis), eIF-4B, Rcc1,* and *Mbs/PPP1R12B* (see also Fig. 5). Expression of the housekeeping genes *Act5C, GAPDH* and *Oda* and two highly conserved RNase P protein co-factor genes *(Rpp30* and *CG14057/Pop5)* are also shown for comparison (blue). C. Heat map showing the expression levels (fragments per kilobase per million mapped reads, FPKM) for the homologs of the same set of *RPR*-recipient genes and housekeeping genes in *T. castaneum* (see Materials and Methods for details and S2 Table for locus information of the genes).
(TIFF)

S2 Fig Sequence alignment of *RPR* genes shows typical *pol III* signals are absent in insects and crustaceans. The RPR-coding region is indicated in uppercase and flanking regions are in lowercase. A. Alignment of *RPR* genes from 29 insect and crustacean species. All lack a PSE element and an appropriately positioned TATA box. About 40% of these genes have a poly-T stretch (red) but the position relative to the 3′-end of RPR is variable suggesting that they are unlikely to be *bona fide* pol III terminators. B. Alignment of *RPR* genes from 14 species in various phyla/superphyla (Arthropoda, Nematoda, Lophotrochozoa, Deuterostomia, and Porifera) highlighting typical pol III signal elements: PSE (blue), TATA box 21-27 nucleotides upstream of RPR (green), and 3′ poly-T stretch of 4-5 nucleotides (red). The arthropods include Chelicerata [*Centuroides sculpturatus* (Arizona bark scorpion); *Ixodes scapularis* (deer tick); *Metaseiulus occidentalis* (predatory mite); *Parasteatoda tepidariorum* (house spider)] and a Myriapoda [*Strigamia maritima* (coastal centipede)]. All *RPR* sequences were obtained from their respective genomes except for the crustacean *Litopenaeus vannamei* (whiteleg shrimp), where an expressed sequence tag (EST) was used [67]. Representative genes are also shown in Fig. 4 and species relationships are shown in the cladogram in Fig. 5.
(PDF)

S3 Fig *D. virilis* RPR co-purifies with RNase P in *D. melanogaster* S2 cells. Using a two-step chromatographic separation (see Materials and Methods), RNase P activity was purified from *D. melanogaster* S2 cells transfected with the R2 reporter gene (Fig. 2A). Results from the activity assays conducted using aliquots of the eluted fractions from the second step are shown. RNA isolated from these fractions was then subjected to RT-PCR using RPR-specific primers. Products corresponding to the expected RPR size from *D. melanogaster* and *D. virilis* were detected in the same fractions that showed maximal RNase P activity. *D. virilis* RPR was less abundant, either because expression from the transgene is lower and/or due to the possibility that the assembly of the *D. virilis* RPR with *D. melanogaster* RPPs to form the heterologous holoenzyme RNP complex is less efficient than with the endogenous *D. melanogaster* RPR. S, substrate without enzyme; PC, positive control with *in vitro* reconstituted *E. coli* RNase P; IP, input; FT, flow through; W1 and W2, washes.
(TIFF)

S4 Fig RPR is produced as part of a reporter gene with a *UAS-Hsp70 pol II* promoter. The RPR-coding intron from *D. virilis* is sufficient for RPR expression when embedded in an *RFP* reporter gene under the control of the *Act5C* pol II promoter (Fig. 2A). Here we tested another pol II promoter—the *UAS-Hsp70* promoter, which is regulated by Gal4. A. Schematic of the *UAS-Hsp70 -RFP* reporter gene with the *D. virilis* intron. The reporter gene was expressed in *D. melanogaster* S2 cultured cells that also express *Act5C-Gal4*. B. RNA from cells either untransfected (- lane) or transfected (+ lane) with the reporter gene was examined by northern analysis using a probe specific to *D. virilis* or *D. melanogaster* RPR. *D. virilis* RPR was detected only in the transfected cells consistent with expression from the *UAS-Hsp70* promoter. U6 RNA was used as a loading control.
(TIFF)

S5 Fig Secondary structure of selected insect RPRs. The secondary structures of RPR from six insect species are shown. Mfold [48] and sequence alignment were used to predict the structures. Nucleotides conserved among eukaryotes are shown in dark circles.
(TIFF)

S6 Fig *RPR* genes in crustaceans are embedded in *pol II* recipient genes. A. The Diplostraca *Daphnia pulex* (water flea) has ten *RPR* genes (the E-value and score from Infernal are shown [68]). *RPR7* is expressed and may encode the functional RPR [24]. In all cases, *RPR* lacks pol III signals (see also S2 Fig. for *RPR7)*. Scaffolds in the current genomic assembly that are smaller than 5 kb are shown as fragments. B. The Copepoda *Lepeophtheirus salmonis* (salmon louse) and *Eurytemora affinis* (calanoid copepod) each have two *RPR* genes, both of which lack pol III regulatory elements (see also Fig. 4 for *RPR1* in *E. affinis*).
(PDF)

S7 Fig Identification of recipient genes in unannotated genomes. A. Top panel, genomic locus of *D. melanogaster ATPsynC*/*CG1746* showing *RPR* in the last intron. Bottom panel, VISTA nucleotide conservation plot of *D. melanogaster ATPsynC*/*CG1746* compared with the *ATPsynC* loci from the scarce chaser (*L. fulva;* Odonata) and the mayfly (*E. danica;* Ephemeroptera) (colored as in Fig. 1A). The *RPR* genes and the flanking exons are conserved. The conservation of the sequence and site of insertion between species in the basal Odonata and Ephemeroptera with the derived Dipteran *(Drosophila)* species suggests the insertion event occurred in a common ancestor of the insects. B. Intron-exon map of the *eIF-4B* gene in species of Lepidoptera, Coleoptera and Hemiptera. With the exception of *O. taurus* (Coleoptera) and *D. citri* (Hemiptera), *RPR* genes are present in introns that separate the same exons in these species. The predicted intron-exon arrangement of the genes was determined using tBLASTx [69] and GeneScan [70]. There is limited nucleotide conservation outside the *RPR* gene and homology between exons was determined using the encoded amino acid sequences. Exons encoding comparable blocks of amino acids are connected by lines (green). Alignment of the eIF-4B N-terminal sequences is shown in the bottom panel. Intron-exon junctions are indicated by a bar (⌶); a black arrow indicates the RPR-coding intron in *H. melpomene*, *B. mori*, *A. planipennis, L. decemlineata and A. pisum*.
(TIFF)

S8 Fig Sequence alignment of *MRP RNA* genes shows typical pol III signals in insects. Alignment of the MRP RNA-coding regions (uppercase) and flanking sequences (lowercase) shows a PSE element (blue), an appropriately positioned TATA box (green) and a poly-T stretch (red) in each gene. The *MRP RNA* genes were identified using Infernal and a covariance model built from previously identified *MRP RNA* genes [14]. All identified sequences were verified to have both the mCR-I and mCR-V as well as the “GARAR” consensus in L8 [14], [17].
(PDF)

S1 Table Sequence databases for genomic analysis. Genomic sequences as scaffolds, contigs or complete assemblies were downloaded in FASTA format and used for identification of *RPR*, *MRP*, *U6* and *7SK* RNA genes using Infernal 1.1 [47]. SAMtools [55] was used to isolate flanking regions of identified genes.
(DOCX)

S2 Table Publicly available RNA-seq and ChIP datasets used. The RNA-seq data were indexed and sorted using SAMtools [55] and visualized using the Integrative Genomics Viewer [54]. RPR is not a polyadenylated transcript, but it is found in RNA-seq analysis at similar levels in both polyA^+^-selected and total RNA samples (Fig. 1C). ChIP data from the Berkley Drosophila Transcription Network Project (BDTNP) was visualized using the Integrative Genomics Viewer (Fig. 1D). RPKM values were calculated from the RNA-seq data with Cufflinks [60] and used to generate a heat map (S1 Fig.). References: [52], [63], [64], [71]–[73]
(DOCX)

S3 Table Genomic information for the *T. castaneum* recipient gene (*Mbs/PPP1R12B*), *RPRs*, and orthologs of recipient genes in other species. The gene IDs and the alternate IDs in Tcas3.22 (EMBL) identified by a TBLASTN search [61] are shown. Cufflinks was used to determine the locus and gene span [60]. Reference housekeeping genes are shown in blue.
(DOCX)
