# Supplementary figures and images for: Transcriptional Control of an Essential Ribozyme in Drosophila Reveals an Ancient Evolutionary Divide in Animals
Source: PLoS Genet. 2015 Jan 8;11(1):e1004893. doi: 10.1371/journal.pgen.1004893 (PMC4287351; doi:10.1371/journal.pgen.1004893)

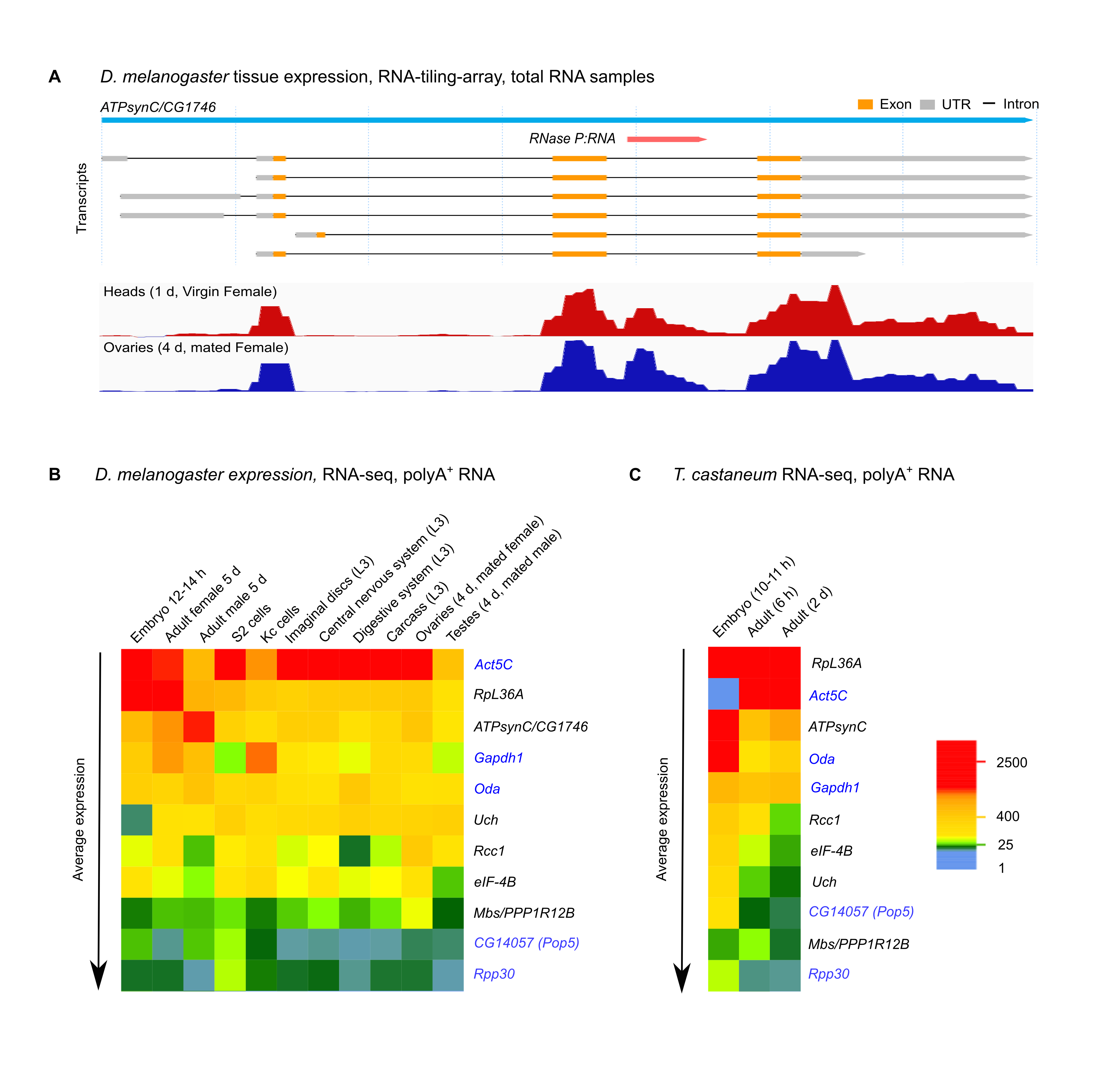

Supplement: S1 Fig — Expression of RPR, ATPsynC/CG1746 and homologs of other RPR-recipient genes in D. melanogaster and T. castaneum. A. Expression of ATPsynC/CG1746 and RPR in heads of 1-day-old virgin females and ovaries of 4-day-old mated females. Samples were total RNA analyzed for expression levels with tiling arrays [52]. B. Heat map showing expression of the indicated genes in D. melanogaster embryos, adults (male and female), S2 or Kc tissue culture cells, and various tissues (imaginal discs, central nervous system, digestive system and carcass of third instar larva (L3) as well as ovaries and testes of 4-day-old adults). ATPsynC/CG1746 is expressed at high levels in D. melanogaster–the average expression level makes it one of the top 200 most highly expressed genes. Expression levels (reads per kilobase per million mapped reads, RPKM) for the D. melanogaster homologs of five RPR-recipient genes in other animals are shown: RpL36A, Uch (Ubiquitin carboxyl-terminal hydrolase 1, USP43 in N. vitripennis), eIF-4B, Rcc1, and Mbs/PPP1R12B (see also Fig. 5). Expression of the housekeeping genes Act5C, GAPDH and Oda and two highly conserved RNase P protein co-factor genes (Rpp30 and CG14057/Pop5) are also shown for comparison (blue). C. Heat map showing the expression levels (fragments per kilobase per million mapped reads, FPKM) for the homologs of the same set of RPR-recipient genes and housekeeping genes in T. castaneum (see Materials and Methods for details and S2 Table for locus information of the genes). (TIFF) [file pgen.1004893.s001.tif]

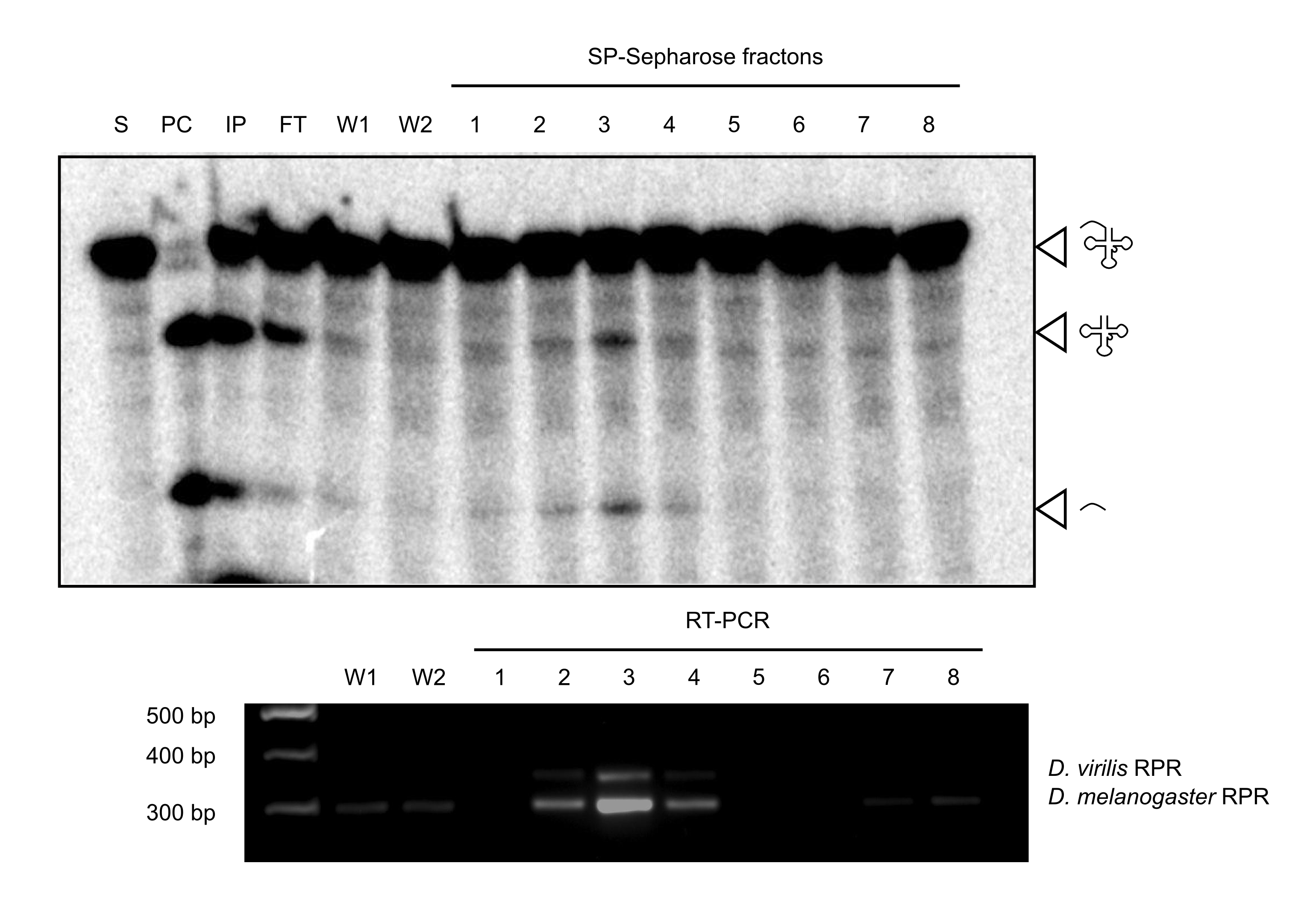

Supplement: S3 Fig — D. virilis RPR co-purifies with RNase P in D. melanogaster S2 cells. Using a two-step chromatographic separation (see Materials and Methods), RNase P activity was purified from D. melanogaster S2 cells transfected with the R2 reporter gene (Fig. 2A). Results from the activity assays conducted using aliquots of the eluted fractions from the second step are shown. RNA isolated from these fractions was then subjected to RT-PCR using RPR-specific primers. Products corresponding to the expected RPR size from D. melanogaster and D. virilis were detected in the same fractions that showed maximal RNase P activity. D. virilis RPR was less abundant, either because expression from the transgene is lower and/or due to the possibility that the assembly of the D. virilis RPR with D. melanogaster RPPs to form the heterologous holoenzyme RNP complex is less efficient than with the endogenous D. melanogaster RPR. S, substrate without enzyme; PC, positive control with in vitro reconstituted E. coli RNase P; IP, input; FT, flow through; W1 and W2, washes. (TIFF) [file pgen.1004893.s003.tif]

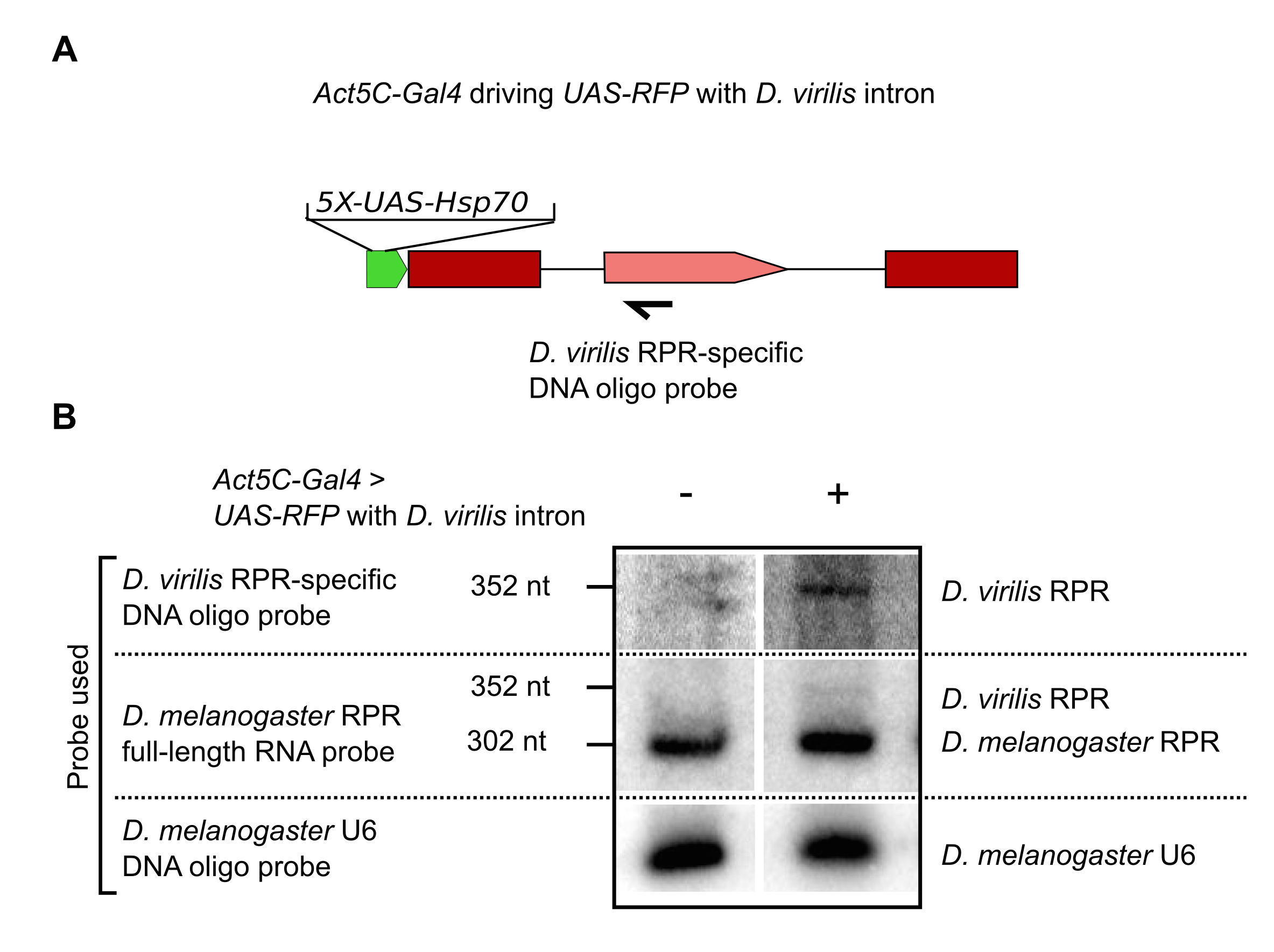

Supplement: S4 Fig — RPR is produced as part of a reporter gene with a UAS-Hsp70 pol II promoter. The RPR-coding intron from D. virilis is sufficient for RPR expression when embedded in an RFP reporter gene under the control of the Act5C pol II promoter (Fig. 2A). Here we tested another pol II promoter—the UAS-Hsp70 promoter, which is regulated by Gal4. A. Schematic of the UAS-Hsp70 -RFP reporter gene with the D. virilis intron. The reporter gene was expressed in D. melanogaster S2 cultured cells that also express Act5C-Gal4. B. RNA from cells either untransfected (- lane) or transfected (+ lane) with the reporter gene was examined by northern analysis using a probe specific to D. virilis or D. melanogaster RPR. D. virilis RPR was detected only in the transfected cells consistent with expression from the UAS-Hsp70 promoter. U6 RNA was used as a loading control. (TIFF) [file pgen.1004893.s004.tif]

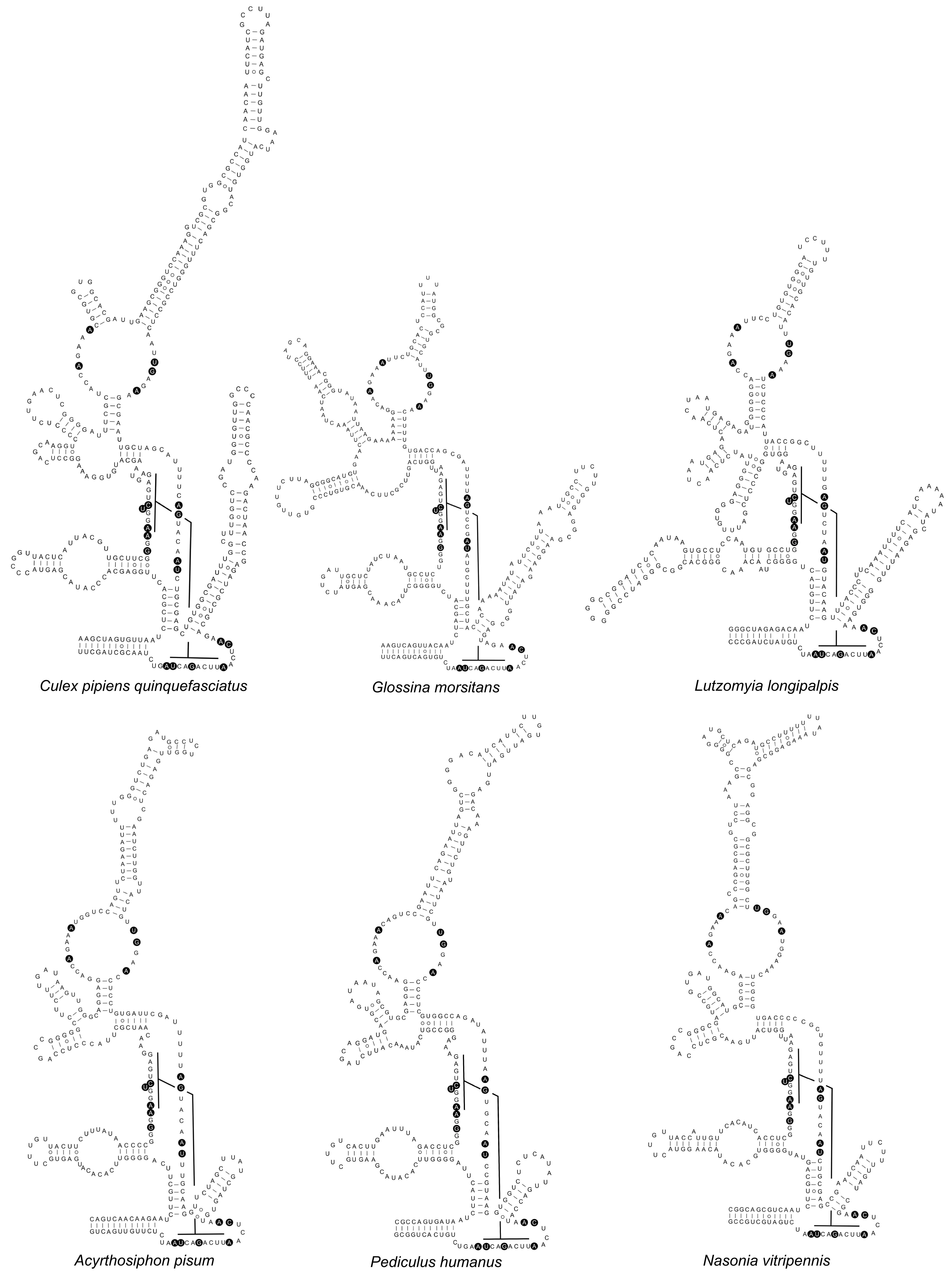

Supplement: S5 Fig — Secondary structure of selected insect RPRs. The secondary structures of RPR from six insect species are shown. Mfold [48] and sequence alignment were used to predict the structures. Nucleotides conserved among eukaryotes are shown in dark circles. (TIFF) [file pgen.1004893.s005.tif]

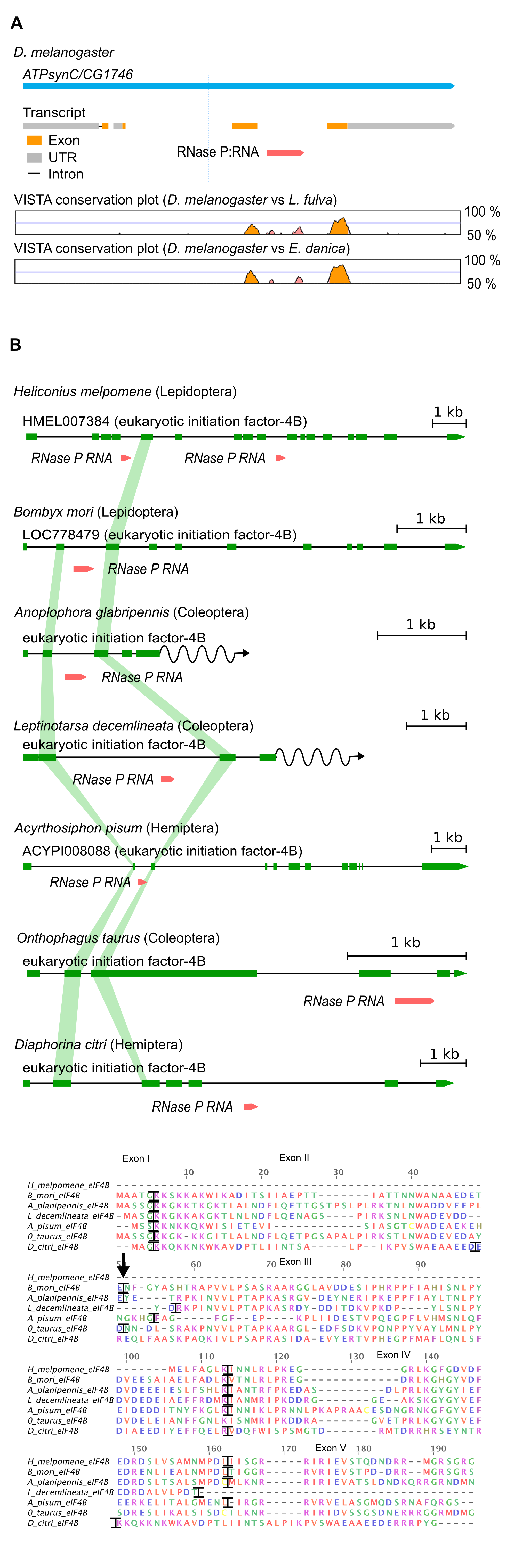

Supplement: S7 Fig — Identification of recipient genes in unannotated genomes. A. Top panel, genomic locus of D. melanogaster ATPsynC/CG1746 showing RPR in the last intron. Bottom panel, VISTA nucleotide conservation plot of D. melanogaster ATPsynC/CG1746 compared with the ATPsynC loci from the scarce chaser (L. fulva; Odonata) and the mayfly (E. danica; Ephemeroptera) (colored as in Fig. 1A). The RPR genes and the flanking exons are conserved. The conservation of the sequence and site of insertion between species in the basal Odonata and Ephemeroptera with the derived Dipteran (Drosophila) species suggests the insertion event occurred in a common ancestor of the insects. B. Intron-exon map of the eIF-4B gene in species of Lepidoptera, Coleoptera and Hemiptera. With the exception of O. taurus (Coleoptera) and D. citri (Hemiptera), RPR genes are present in introns that separate the same exons in these species. The predicted intron-exon arrangement of the genes was determined using tBLASTx [69] and GeneScan [70]. There is limited nucleotide conservation outside the RPR gene and homology between exons was determined using the encoded amino acid sequences. Exons encoding comparable blocks of amino acids are connected by lines (green). Alignment of the eIF-4B N-terminal sequences is shown in the bottom panel. Intron-exon junctions are indicated by a bar (⌶); a black arrow indicates the RPR-coding intron in H. melpomene, B. mori, A. planipennis, L. decemlineata and A. pisum. (TIFF) [file pgen.1004893.s007.tif]
