# Supplementary material for: Transcriptional Control of an Essential Ribozyme in Drosophila Reveals an Ancient Evolutionary Divide in Animals
Source: PLoS Genet. 2015 Jan 8;11(1):e1004893. doi: 10.1371/journal.pgen.1004893 (PMC4287351; doi:10.1371/journal.pgen.1004893)
Supplement: S2 Fig — Sequence alignment of RPR genes shows typical pol III signals are absent in insects and crustaceans. The RPR-coding region is indicated in uppercase and flanking regions are in lowercase. A. Alignment of RPR genes from 29 insect and crustacean species. All lack a PSE element and an appropriately positioned TATA box. About 40% of these genes have a poly-T stretch (red) but the position relative to the 3′-end of RPR is variable suggesting that they are unlikely to be bona fide pol III terminators. B. Alignment of RPR genes from 14 species in various phyla/superphyla (Arthropoda, Nematoda, Lophotrochozoa, Deuterostomia, and Porifera) highlighting typical pol III signal elements: PSE (blue), TATA box 21-27 nucleotides upstream of RPR (green), and 3′ poly-T stretch of 4-5 nucleotides (red). The arthropods include Chelicerata [Centuroides sculpturatus (Arizona bark scorpion); Ixodes scapularis (deer tick); Metaseiulus occidentalis (predatory mite); Parasteatoda tepidariorum (house spider)] and a Myriapoda [Strigamia maritima (coastal centipede)]. All RPR sequences were obtained from their respective genomes except for the crustacean Litopenaeus vannamei (whiteleg shrimp), where an expressed sequence tag (EST) was used [67]. Representative genes are also shown in Fig. 4 and species relationships are shown in the cladogram in Fig. 5. (PDF) [file pgen.1004893.s002.pdf]

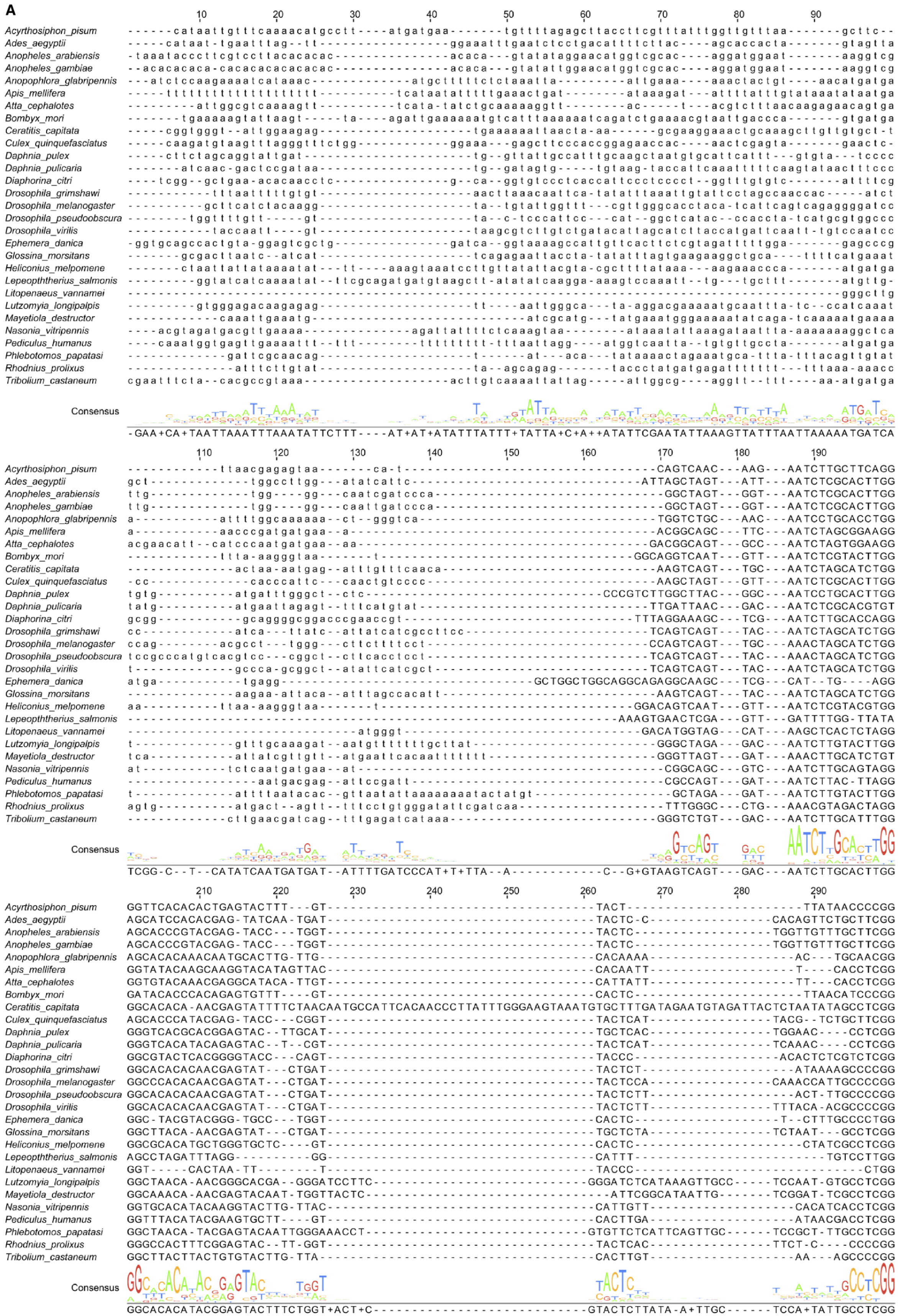
A
Acyrthosiphon_pisum
Ades_aegyptii
Anopheles_arabiensis
Anopheles_gambiae
Anopophlora_glabripennis
Apis_mellifera
Atta_cephalotes
Bombyx_mori
Ceratitis_capitata
Culex_quinquefasciatus
Daphnia_pulex
Daphnia_pulicaria
Diaphorina_citri
Drosophila_grimshawi
Drosophila_melanogaster
Drosophila_pseudoobscura
Drosophila_virilis
Ephemera_danica
Glossina_morsitans
Heliconius_melpomene
Lepeopththerius_salmonis
Litopenaeus_vannamei
Lutzomyia_longipalpis
Mayetiola_destructor
Nasonia_vitripennis
Pediculus_humanus
Phlebotomos_papatasi
Rhodnius_prolixus
Tribolium_castaneum
Consensus

```
                            310       320       330       340       350       360       370       380       390
Acyrthosiphon_pisum         GAAGGTCTGAGGCAAAATC------GCT-----------------------------------------------------------------TA-CCCT
Ades_aegyptii               GAAGGTCTGAGAG-GAAG---------CAATTT--------------------------------------------------------------ATGTC
Anopheles_arabiensis        GAAGGTCTGAGAT-AAGG---------CATGCGAA-----------------------------------------------------------TATGCC
Anopheles_gambiae           GAAGGTCTGAGAT-AAGG---------CATGCGAA-----------------------------------------------------------TATGCC
Anopophlora_glabripennis    GAAGGTCTGAGGCAGG--C------AAC-----------------------------------------------------------------TACC-CC
Apis_mellifera              GAAGGTCTGAGATTAAACC------AAT-----------------------------------------------------------------AAACATT
Atta_cephalotes             GAAGGTCTGAGATAAGATT------GTT-----------------------------------------------------------------AAACGTT
Bombyx_mori                 GAAGGTCTGAGTAAAAGCCACCAGTGTTTTGTG------------------------------------------------------------TA-CATT
Ceratitis_capitata          GAAGGTCTGAGAGTTGGTC---AAAGTTCATT-----TCA-------AACATG--CTGTGGTG-----------CGTGTGAACTTAAACCGATGCATTTT
Culex_quinquefasciatus      GAAGGTCTGAGAG-TAAG---------CATGTGGA-----------------------------------------------------------AAGGCC
Daphnia_pulex               GAAGGTCTGAGACGAGG---------CTTTT------------------------------------------CTCATTGTCGGAGAGTG-----ATGCT
Daphnia_pulicaria           GAAGGTCTGAGAAAAAG---------CTGTG------------------------------------------CTCTTGTTTGTAGAGTTC---CATGTT
Diaphorina_citri            GAAGGTCTGAGTTTTGGAT-------TCAAATGT-------------------------------------------------------------ACTTT
Drosophila_grimshawi        GAAGGTCTGAGAATTGGTC---AAAGCTCCCTTGT--TTGTGTGCACAACAAGCGATTTGCTGTTAAAGGTGATCGTTTGTCGCGGAGT---TTCATTTC
Drosophila_melanogaster     GAAGGTCTGAGAATCGGCC---GA-GCCAGCT-GT--TTGT---TGC---------------------------------------GGC---TTCATTTC
Drosophila_pseudoobscura    GAAGGTCTGAGATTTGGCC---AA-GCCTCCTTGT-GTTGT---TACAACATGCAGTGCATCG--AGAGGTGCCTGTGTGTCG-GAGGC---TTAATTTC
Drosophila_virilis          GAAGGTCTGAGATATGGTC---AAAGCTCCCTTGTTGCTGT---AACAACATACAAAA-GCCG--AGAGGTGATTGTGTGTCGCGGAGC---TTCATTTT
Ephemera_danica             GAAGGTCTGAGG--TAGGG-------CCAACTG--------------------------------------------------------------ACACT
Glossina_morsitans          GAAGGTCTGAGAATTGGTC---AAAGTTCGCT-----TCA-------AACGTGTCCCGTGTTGTTTCTTAGGGGCATGTGAACTTAAACTAATCAATTTC
Heliconius_melpomene        GAAGGTCTGAGTAAAGGTC-TAATTGTTCTTTA------------------------------------------------------------TA-CATT
Lepeophtherius_salmonis     GAAGGTCTGAGAGAAGAGC-----CG-C--ATA------------------------------------------------------------TA---CC
Litopenaeus_vannamei        GAAGGTCTGAGAG-AGGAG-------TCATGT---------------------------------------------------------------ATGCT
Lutzomyia_longipalpis       GAAGGTCTGAGATAAGGT-----GGGCCCTCG-------------------------------------------ATTTGTGGGAGGGT---TTA-TGTT
Mayetiola_destructor        GAAGGTCTGAGAAAAGGT-----GTGCTTCAT-------------------------------------------GTTGAAGTGTGTGT---TTAACATT
Nasonia_vitripennis         GAAGGTCTGAGATAAGGTC------ATT-----------------------------------------------------------------GAACGCT
Pediculus_humanus           GAAGGTCTGAGA-AAGGGC-----CGTC--ATA------------------------------------------------------------TAACATT
Phlebotomos_papatasi        GAAGGTCTGAGTGAAGGT-----GGATTCTCG-------------------------------------------GTCTG--GGAGGGT---CTA-TGTT
Rhodnius_prolixus           GAAGGTCTGAGATCAGGA--------CTGTCTTTC-----------------------------------AACCTCTTAATTGGAGGGT--------GTT
Tribolium_castaneum         GAAGGTCTGAGATGTG--T------CTT-----------------------------------------------------------------TAATGCT
Consensus                   GAAGGTCTGAGATAAGGTC---AAAG+TATCTGGT-GTTGT---TACAACATGCA+TGTG+TGTTAGAGGTG+CTGTTTGTCGGAGAGTT-ATTAATGTT

                            410       420       430       440       450       460       470       480       490
Acyrthosiphon_pisum         CCAGCCGGGGGCCTTCT---TAATAAAGTTGGGCAGGAGAACCAGAAA---TTGGT---CCAGTTCTAA-------------------------GATTTT
Ades_aegyptii               TCAGCAAGACAGCCTTTT---GA--CAGGGGCGTTGTACAACTAGAAA---TGCAGTGTGCGTCTTGGA-----TTTG-----GAGTTGT----AGTGTC
Anopheles_arabiensis        TCAGCAAGGCAGCTCTTT---GAG--AAGAGCGTT-CGCTACCAGCAA---TACATCA---GTTGTGGA-------TG--------ACGC----AGTGTC
Anopheles_gambiae           TCAGCAAGGCAGCTCTTT---GAG--AAGAGCGTT-CGCTACCAGCAA---TACATCA---GTTGTGGA-------TG--------ACGC----AGTGTC
Anopophlora_glabripennis    CTAGCTGGGGGCTCGC------ATATTGCGAGACAAGGGAACCAGAAA---TATGT---CAAC--GAAG-------------------------CATCAA
Apis_mellifera              CTAGACGGATGATACC------ATAAGGGTATGTACGGGAACCAGAAA---TAGCT---TTA---CGTT-------------------------GC-CAA
Atta_cephalotes             CTAGCCGGACGGCATC------ATAATGATGCGTACGAGAACCAGAAA---TAGCT---TTG---CGTT-------------------------TTTCAA
Bombyx_mori                 CTAGCCGGATGCTTCCT---TAACAAGGATGAAGAGG-GAACCAGAAA---TTA------TGGTACAAT-------------------------TTCT--
Ceratitis_capitata          CTAGCAGGAAACTTGTAT-----GATTAAGAGGCGAAGGGACAAGAAA---TTC------TGAAA-----------------------------------
Culex_quinquefasciatus      TCAGCAAGGTCCCCTCTT---GAACTCGGGGGATTTCGCTACCAGAAAACGTGCGTGGCACGATTTGAAGCGGGTCTGTCCAAAGGTCGT----CGTGGC
Daphnia_pulex               CTAGCCGGGCAGCCATTT-----CGCGAAGGCGCAGGGGAATAGAAAG---AATATCGATTGGAGTGTC-------------------------GTTTAG
Daphnia_pulicaria           CTAGCCGGACAACCTTCT-----TGCGAAGGTGCAGGGGAATAGAAAG---AGTATCGAATGGAATGTT-------------------------ATCTG-
Diaphorina_citri            CTAGCCGGAAGTCTCTCT---CACCAGAGAGAGTGAGGGAACCAGAAA-----TAGCGCACGCTCCGGGC---------------CCCATT--TAAACCC
Drosophila_grimshawi        CCAGCAGGAAACCTGTGT-----GATCGCAGGGCGAAAGTACCAGAAA---TCC------TGCCTCGCT-------------------------GCT---
Drosophila_melanogaster     CCAGCAGGAAACCTGTGT-----GATTGCAGGGCGAAAGTACCAGAAA---TCC------TGCTACCAG-------------------------GT----
Drosophila_pseudoobscura    CTAGCAGGAAACCTGTGT-----GATTGCAGGACGAAAGTACCAGAAA---TAC------TGCGGCTTG-------------------------GC----
Drosophila_virilis          CTAGCCGGAAACTTGTGT-----AATCACAAGGCGAAAGTACCAGAAA---TGC------TGCAGTATG-------------------------GCT---
Ephemera_danica             CTAGCAGGGTGCCCCCAT---AGTTTAGGGGGGTGGGGGAGCAGAAAA-----T-------------------------------CCTGTC--TAGTGGC
Glossina_morsitans          CTAGCAGGAAACTTGTAT-----AATTAAGAGGCAAAAGGACAAGAAA---TTC------TGCA------------------------------------
Heliconius_melpomene        CTAGCCGGATGCTTTCT---TTATAAGAAAGGCAAGG-GAACCAGAAA---TTA------CAATGCCAA-------------------------GTTT--
Lepeophtherius_salmonis     CTAGCAGGGTACTTTCTTTGTTATAAGGAAGAGAAGG--GATCAGAAA---CAAGG---TCAAAGTGAG-------------------------TATGCA
Litopenaeus_vannamei        CTTGCAGGGCAACCTCCA---AA---AGGAGGGACAGGACAACAGAAAG---GCCCTG-----ACTGGA--------G-------GTCATCAAGGACACC
Lutzomyia_longipalpis       CTAGCAGGACACTCAACT-----AATGAGA-GATGGGGGGACCAGAAA---TTC------CTGTGT--------------------------------GG
Mayetiola_destructor        CTAGCTGGATGCTTTTAT-----AGTTAGAAGGTGGGGGGACCAGAAA---TTC------TGTTATTAT-------------------------TTTAAT
Nasonia_vitripennis         CCAGCCGGGCGATGCC------GTGATGGCATGCGCGAGAACCAGAAA---CAGCC---CGAG--CGCG-------------------------TCCTAA
Pediculus_humanus           CTAGCAGGATGACGT----GATAATAGCGTGCGAGGG--AACCAGAAA---CA-GT---CCGAATTCAG-------------------------AAT---
Phlebotomos_papatasi        CTAGCTGGACACTTAATT-----AACAAGA-GATAGGGGGACCAGAAA---TTT------CTGTGC--------------------------------A-
Rhodnius_prolixus           CC--CTAAATAA---------------GGGGCGCGGGGTAACAGAAAA---AGCA----ATGACATATC-------------------------CTTGG-
Tribolium_castaneum         CCAGAAGGGCGCTTGC------ATTTTGCAAGGCGAGAACACCAGAAA---TCTG----CGAT--GAGG-------------------------TTTCAA
Consensus                   CTAGCAGGACACCTTTTT-GT+ATAT+GGAGGGCGGGGGAACCAGAAA---TACATCG+CTG+TGTGAG-----T-TG-------GTCGTC--TGTTGAC

                            510       520       530       540       550       560       570       580       590
Acyrthosiphon_pisum         TGGGTCTGAGATGCCT-------CTG-------GTTGAGAC-------TACAATCT-----TGGTTACTGT-----------------TGGAAC--CTCC
Ades_aegyptii               CCAATCTGGGTTTC----CCGGGGATA--CTC-----AAGA---ATGCCCGATTTC----ACTACAGTTGC-CGATTCC----CAAGTTGGAA--ATGTA
Anopheles_arabiensis        CTGGCC---GCTAC----------------TT----GAAAG---GTG-CAAACCTC----TCGGTTTCGGCTCGGGCAC----TGATTGAAAACAGCGGA
Anopheles_gambiae           CTGGCC---GCTTC----------------TT----GAAAG---GTG-CAAACCTC----TCGGTTTCGGCTCGGGCAC----TGATTGAAAACAGCGGA
Anopophlora_glabripennis    --AGCCAAGGAT-----------T-AC---TTACGT-ATAGG--TGA-TATGCCTA---------GTTGGT-----------------TGGAAG--CCCT
Apis_mellifera              TTAGCCCAGGAAACT--------TCAT---TTGAAGTAGAGG--TACTTGGAAACG---------AAAAGA-----------------TGGAAA--CCCG
Atta_cephalotes             AATGCTGAGGACGCT--------TTTT---TAAAAGCAGAGG--CGACTGGAAACG---------TGAAGT-----------------TGAAAT--CTCG
Bombyx_mori                 --AGTCTAGGATTCCTT----GTCTGT---GTTGATAGGGAA--GAGACATAGTTA-----TTGTTACTAT-----------------TGGAAA--CCCT
Ceratitis_capitata          ATTCCAATAGGTTTT---------------------AGGAC---------CTTACG----AATATT--CAT-----------------TGAAAT--CCTT
Culex_quinquefasciatus      GCCATCAACAATTCATCGCCTTAGATGAGCTTGTTGGAATC---ATGGTGTATCGG----ACGACTTTGGC-CAGGTCCGCCTCAATTGAGAA--GCGAA
Daphnia_pulex               ACCCATGATGTCCATTA--------------------GGACT--AGGCGGAGAACT----CCATTT-CGAT-----------------TGGAACTGCTTC
Daphnia_pulicaria           -CCCATGATGCCCTTTGT-------------------GGGTT--AGGCGTAAACTT----CCAAATACGAT-----------------TGGAACAGCCTC
Diaphorina_citri            CCGTCTGTGAAACC----------------TCCTCGTAGGGCCTGAGACGAAGTCC----GGAGT--GGGC---------------GATGGAACCTCTCA
Drosophila_grimshawi        GTTATTGTTATTGTTGTTG-----------TTGCCAACGACG--ATGACGACGACG----CGCGTTG-CAT-----------------TGAAAT--CTTT
Drosophila_melanogaster     GTTGCCGTTGCCCCC----------------GG----TGAC-------CGCCG-C-----CTGGTTGGCAT-----------------TGAAAC--CTTT
Drosophila_pseudoobscura    GTTGCCATCGGCGCT----------------CGCGTCTGAC-------TGCCG-C-----CTTGTTG-CAT-----------------TGAAAA--CTTT
Drosophila_virilis          GTTGCCG-CACCGCT----------------TGCCGTTTGC-------CGCCGGC-----CATGTTG-CAT-----------------TGAAAA--CTTT
Ephemera_danica             TAGCCTG-GGATCC----------------TCTTTG-AGGG---GAGGTGCAGCCG----CTATT--CAGT-----------------TGAAACCTCCCA
Glossina_morsitans          -CTCCA-----TTTT---------------------ATGGC--------------------GCGTG--CAT-----------------TGGAAA--CTTT
Heliconius_melpomene        --AGCTTGGGATACCT-----AACCGA---GTGGTTAAGGAG--TAGGTGTA-TCC-----TTGGTATTGT-----------------TGGAAA--CTCT
Lepeophtherius_salmonis     -TAACTGTTGATATAAG----TATTTTCAACTTATTGAGG--------TTCTGTGC-----TTTTCTTTGAA----------------TGAAAA--CTCT
Litopenaeus_vannamei        TTGGACACTAATCT-----------------------AAAG---GTGGAGATTCCT----CCAGTCTTGGT----------------TGGGAGTCCTGTG
Lutzomyia_longipalpis       CATCCTTTTGTTGC-----------------------------------------------------ACAT-----------------TGAAAT--CTCC
Mayetiola_destructor        TTTGATTTTGGTGTC---------------------ACAGCC--AAAACGAATTTA----AATAATAACAT-----------------TGAAAT--CTCC
Nasonia_vitripennis         --AGCCGGGGATGCTCAGATGCCTTTT---TTTATAAAGAGG--CGAGCGGAGGCGGCGCTTGGCTTGGGA-----------------TGGAAA--CTCG
Pediculus_humanus           -TAGTCGGGGACATCAT----TCTTGT-GATTGATTGAGA--------CAAAGTTC-----TGTAATTCGT-----------------TGGAAC--CCCT
Phlebotomos_papatasi        -----TTTAG--AC-----------------------------------------------------ACAT-----------------TGAAAT--CTTC
Rhodnius_prolixus           ---CAAGA-GTTTAATGC-------------------GGA-T--AAGTGG--------------TT---GG-----------------TGGGATAGCC-C
Tribolium_castaneum         --AGCCAAGGAT-----------TCGC---TCTGGCGATAGG--TGG-TGGAGCTT---------TATCGT-----------------TGGAAT--TTCT
Consensus                   +T+GCCG+GGATGCTT++CCGTATT+T-G+TTGATT+AGA+G--AAGGCGAAG+C+----CTTGTTTTCGTTCGGG++C----++ATTTGGAA+AGCTCT
```

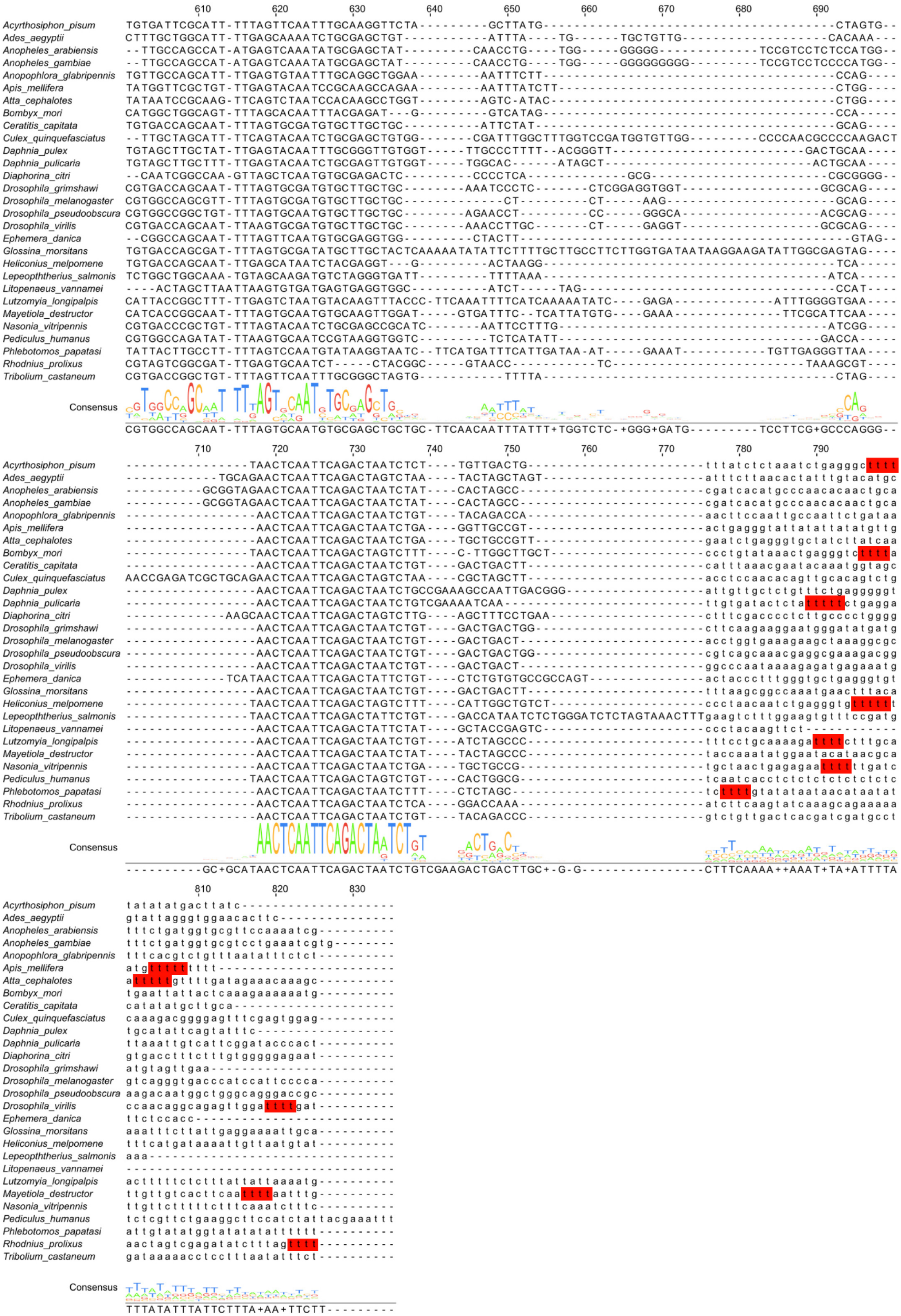
610 620 630 640 650 660 670 680 690
Acyrthosiphon_pisum
Ades_aegyptii
Anopheles_arabiensis
Anopheles_gambiae
Anopophlora_glabripennis
Apis_mellifera
Atta_cephalotes
Bombyx_mori
Ceratitis_capitata
Culex_quinquefasciatus
Daphnia_pulex
Daphnia_pulicaria
Diaphorina_citri
Drosophila_grimshawi
Drosophila_melanogaster
Drosophila_pseudoobscura
Drosophila_virilis
Ephemera_danica
Glossina_morsitans
Heliconius_melpomene
Lepeophtherius_salmonis
Litopenaeus_vannamei
Lutzomyia_longipalpis
Mayetiola_destructor
Nasonia_vitripennis
Pediculus_humanus
Phlebotomos_papatasi
Rhodnius_prolixus
Tribolium_castaneum
Consensus
710 720 730 740 750 760 770 780 790
Consensus
810 820 830
Consensus

B

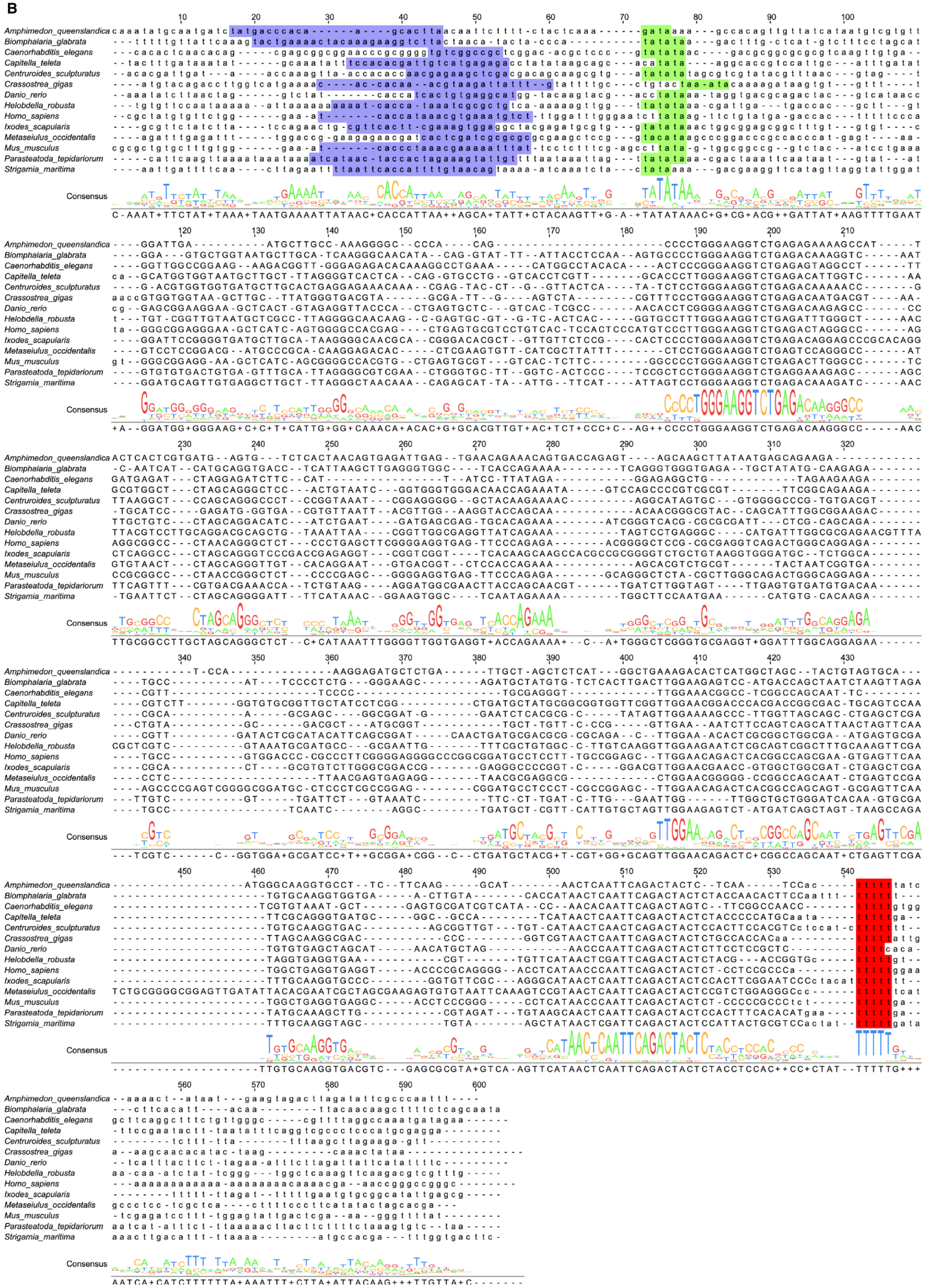
Amphimedon_queenslandica
Biomphalaria_glabrata
Caenorhabditis_elegans
Capitella_teleta
Centruroides_sculpturatus
Crassostrea_gigas
Danio_rerio
Helobdella_robusta
Homo_sapiens
Ixodes_scapularis
Metaseiulus_occidentalis
Mus_musculus
Parasteatoda_tepidariorum
Strigamia_maritima
Consensus
