# Supplementary material for: Transcriptional Control of an Essential Ribozyme in Drosophila Reveals an Ancient Evolutionary Divide in Animals
Source: PLoS Genet. 2015 Jan 8;11(1):e1004893. doi: 10.1371/journal.pgen.1004893 (PMC4287351; doi:10.1371/journal.pgen.1004893)
Supplement: S6 Fig — RPR genes in crustaceans are embedded in pol II recipient genes. A. The Diplostraca Daphnia pulex (water flea) has ten RPR genes (the E-value and score from Infernal are shown [68]). RPR7 is expressed and may encode the functional RPR [24]. In all cases, RPR lacks pol III signals (see also S2 Fig. for RPR7). Scaffolds in the current genomic assembly that are smaller than 5 kb are shown as fragments. B. The Copepoda Lepeophtheirus salmonis (salmon louse) and Eurytemora affinis (calanoid copepod) each have two RPR genes, both of which lack pol III regulatory elements (see also Fig. 4 for RPR1 in E. affinis). (PDF) [file pgen.1004893.s006.pdf]

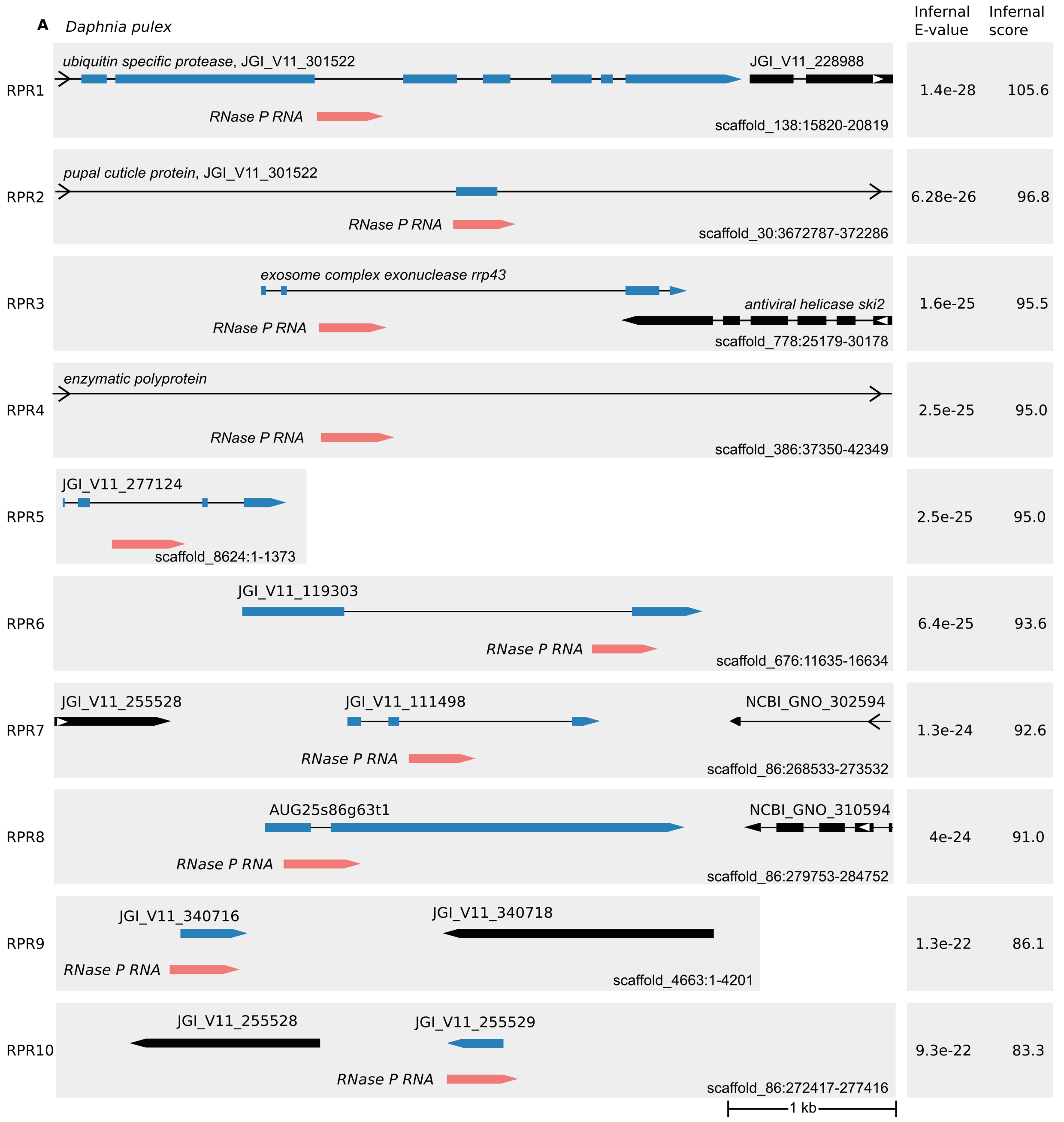

A
Daphnia pulex
Infernal E-value
Infernal score
RPR1
ubiquitin specific protease, JGI_V11_301522
JGI_V11_228988
RNase P RNA
scaffold_138:15820-20819
1.4e-28
105.6
RPR2
pupal cuticle protein, JGI_V11_301522
RNase P RNA
scaffold_30:3672787-372286
6.28e-26
96.8
RPR3
exosome complex exonuclease rrp43
antiviral helicase ski2
RNase P RNA
scaffold_778:25179-30178
1.6e-25
95.5
RPR4
enzymatic polyprotein
RNase P RNA
scaffold_386:37350-42349
2.5e-25
95.0
RPR5
JGI_V11_277124
scaffold_8624:1-1373
2.5e-25
95.0
RPR6
JGI_V11_119303
RNase P RNA
scaffold_676:11635-16634
6.4e-25
93.6
RPR7
JGI_V11_255528
JGI_V11_111498
NCBI_GNO_302594
RNase P RNA
scaffold_86:268533-273532
1.3e-24
92.6
RPR8
AUG25s86g63t1
NCBI_GNO_310594
RNase P RNA
scaffold_86:279753-284752
4e-24
91.0
RPR9
JGI_V11_340716
JGI_V11_340718
RNase P RNA
scaffold_4663:1-4201
1.3e-22
86.1
RPR10
JGI_V11_255528
JGI_V11_255529
RNase P RNA
scaffold_86:272417-277416
9.3e-22
83.3
1 kb

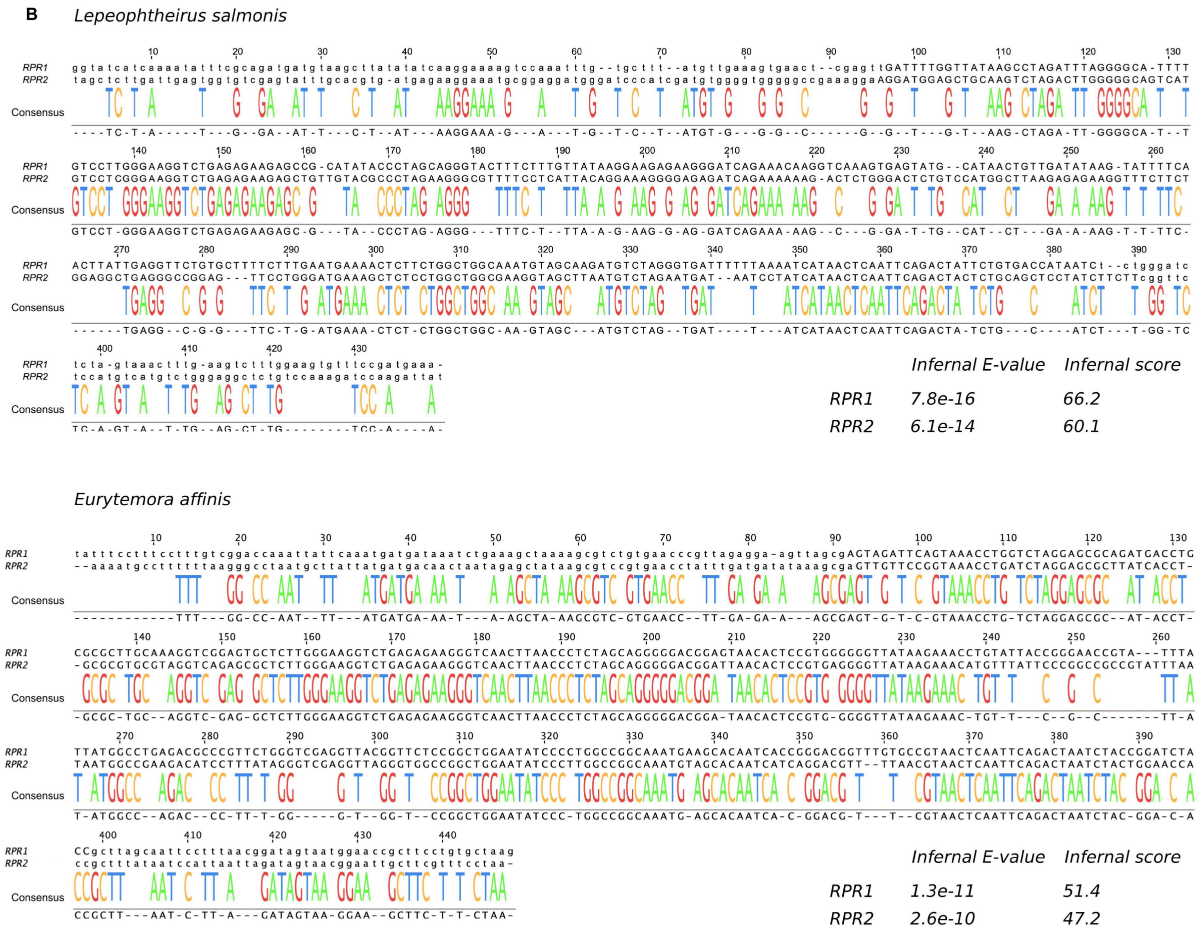
B
Lepeophtheirus salmonis
RPR1
RPR2
Consensus
Infernal E-value
Infernal score
RPR1 7.8e-16 66.2
RPR2 6.1e-14 60.1
Eurytemora affinis
RPR1
RPR2
Consensus
Infernal E-value
Infernal score
RPR1 1.3e-11 51.4
RPR2 2.6e-10 47.2
