# Supplementary material for: Transcriptional Control of an Essential Ribozyme in Drosophila Reveals an Ancient Evolutionary Divide in Animals
Source: PLoS Genet. 2015 Jan 8;11(1):e1004893. doi: 10.1371/journal.pgen.1004893 (PMC4287351; doi:10.1371/journal.pgen.1004893)
Supplement: S8 Fig — Sequence alignment of MRP RNA genes shows typical pol III signals in insects. Alignment of the MRP RNA-coding regions (uppercase) and flanking sequences (lowercase) shows a PSE element (blue), an appropriately positioned TATA box (green) and a poly-T stretch (red) in each gene. The MRP RNA genes were identified using Infernal and a covariance model built from previously identified MRP RNA genes [14]. All identified sequences were verified to have both the mCR-I and mCR-V as well as the “GARAR” consensus in L8 [14], [17]. (PDF) [file pgen.1004893.s008.pdf]

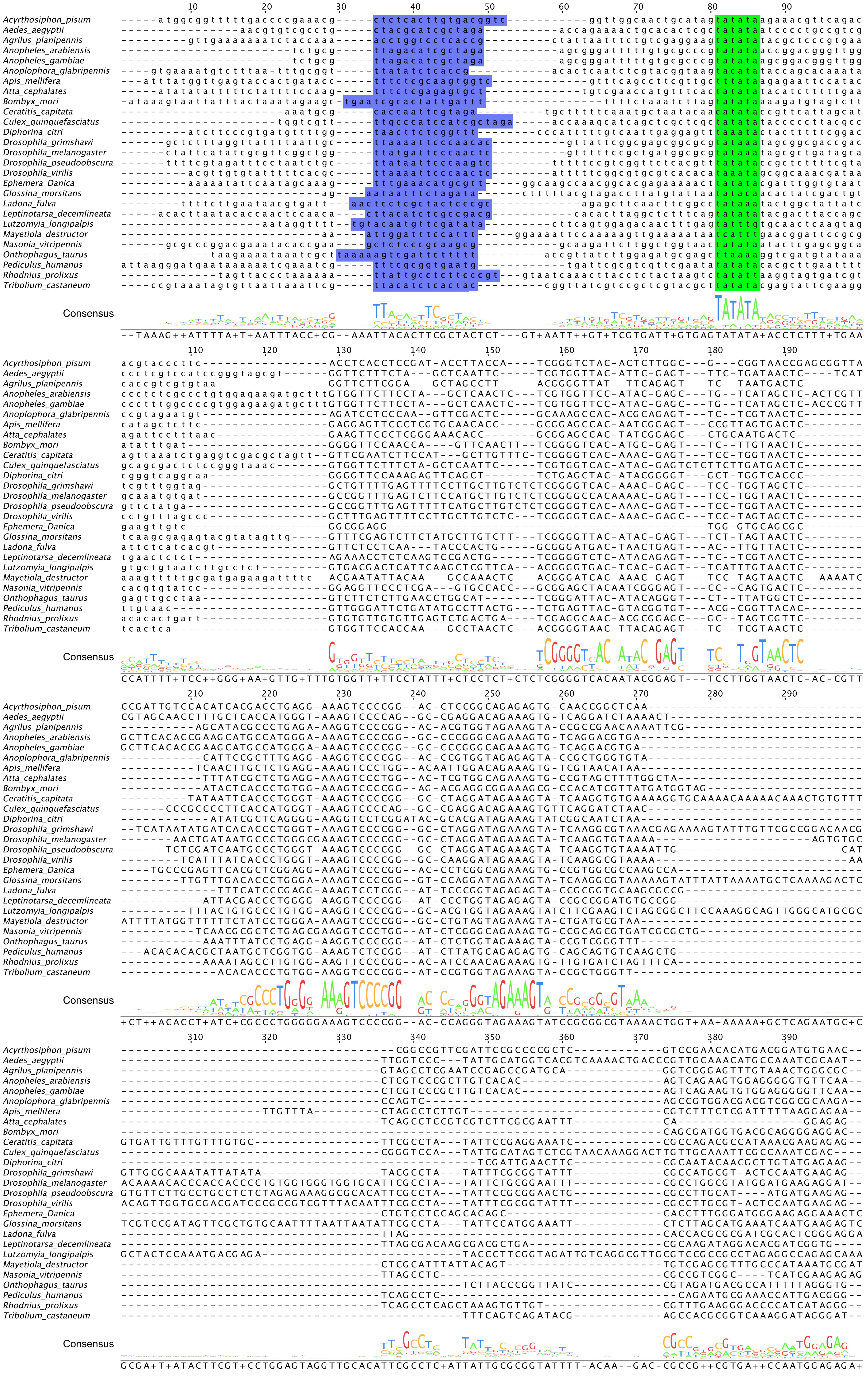

Acyrthosiphon_pisum
Aedes_aegyptii
Agrilus_planipennis
Anopheles_arabiensis
Anopheles_gambiae
Anoplophora_glabripennis
Apis_mellifera
Atta_cephalates
Bombyx_mori
Ceratitis_capitata
Culex_quinquefasciatus
Diphorina_citri
Drosophila_grimshawi
Drosophila_melanogaster
Drosophila_pseudoobscura
Drosophila_virilis
Ephemera_Danica
Glossina_morsitans
Ladona_fulva
Leptinotarsa_decemlineata
Lutzomyia_longipalpis
Mayetiola_destructor
Nasonia_vitripennis
Onthophagus_taurus
Pediculus_humanus
Rhodnius_prolixus
Tribolium_castaneum
Consensus

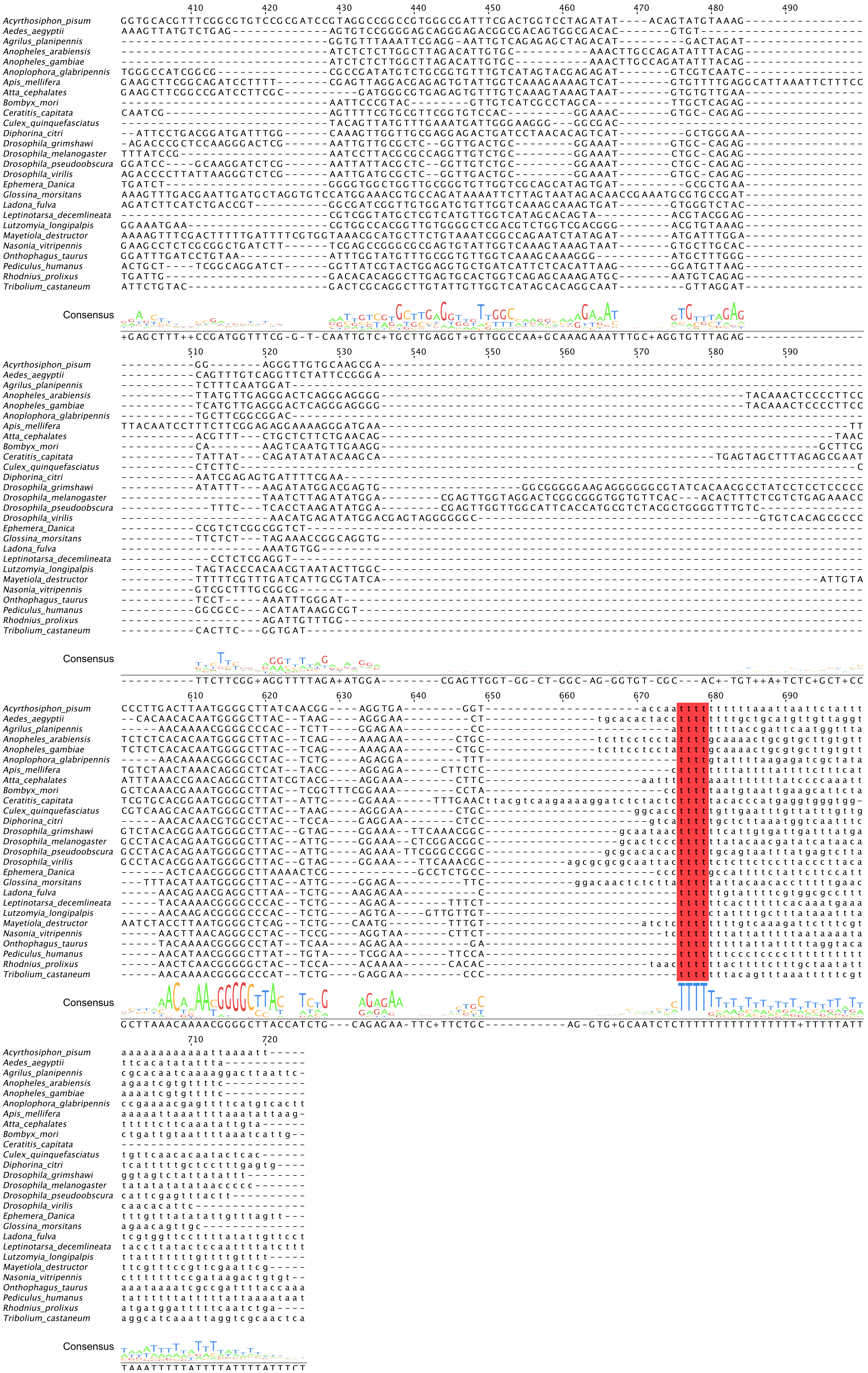
Acyrthosiphon_pisum
Aedes_aegyptii
Agrilus_planipennis
Anopheles_arabiensis
Anopheles_gambiae
Anoplophora_glabripennis
Apis_mellifera
Atta_cephalates
Bombyx_mori
Ceratitis_capitata
Culex_quinquefasciatus
Diphorina_citri
Drosophila_grimshawi
Drosophila_melanogaster
Drosophila_pseudoobscura
Drosophila_virilis
Ephemera_Danica
Glossina_morsitans
Ladona_fulva
Leptinotarsa_decemlineata
Lutzomyia_longipalpis
Mayetiola_destructor
Nasonia_vitripennis
Onthophagus_taurus
Pediculus_humanus
Rhodnius_prolixus
Tribolium_castaneum
Consensus
