# Supplementary material for: Transcriptional Control of an Essential Ribozyme in Drosophila Reveals an Ancient Evolutionary Divide in Animals
Source: PLoS Genet. 2015 Jan 8;11(1):e1004893. doi: 10.1371/journal.pgen.1004893 (PMC4287351; doi:10.1371/journal.pgen.1004893)
Supplement: S1 Table — Sequence databases for genomic analysis. Genomic sequences as scaffolds, contigs or complete assemblies were downloaded in FASTA format and used for identification of RPR, MRP, U6 and 7SK RNA genes using Infernal 1.1 [47]. SAMtools [55] was used to isolate flanking regions of identified genes. (DOCX) [file pgen.1004893.s009.docx]

**Table S1. Sequence databases for genomic analysis.**

| **Organism** | **Database** | **GenBank assembly ID or other IDs** |
| --- | --- | --- |
| *Drosophila melanogaster* | Flybase and NCBI | GCA_000001215.2 |
| *Drosophila pseudoobscura* | Flybase and NCBI | GCA_000001765.2 |
| *Drosophila virilis* | Flybase and NCBI | GCA_000005245.1 |
| *Drosophila grimshawi* | Flybase and NCBI | GCA_000005155.1 |
| *Glossina morsitans* | Vectorbase | GmorY1 |
| *Ceratitis capitata* | i5K pilot project (BCM-HGSC)/NCBI | GCA_000347755.1 |
| *Mayetiola destructor* | i5K pilot project (BCM-HGSC) | Mdes_1.0 (Mdes20100623.genome.fa) |
| *Aedes aegypti* | Vectorbase | AaegL3 |
| *Culex quinquefasciatus* | Vectorbase | CpipJ2 |
| *Anopheles gambiae* | Vectorbase | AgamP4 |
| *Anopheles arabiensis* | Vectorbase | AaraD1 |
| *Lutzomyia longipalpis* | Vectorbase | LlonJ1 |
| *Phlebotomus papatasi* | Vectorbase | PpapI1 |
| *Bombyx mori* | NCBI | GCA_000151625.1 |
| *Heliconius melpomene* | EnsemblMetazoa | Hmel1.23 |
| *Agrilius planipennis* | i5K pilot project (BCM-HGSC)/NCBI | GCA_000699045.1 |
| *Anoplophora glabripennis* | i5K pilot project (BCM-HGSC)/NCBI | GCA_000390285.1 |
| *Tribolium castaneum* | EnsemblMetazoa | GCA_000002335.2 |
| *Apis mellifera* | BeeBase and NCBI Genomes | GCA_000002195.1 |
| *Atta cephalotes* | EnsemblMetazoa Genomes | GCA_000143395.2 |
| *Nasonia vitripennis* | NCBI Genome | GCA_000002325.2 |
| *Diaphorina citri* | i5K pilot project (BCM-HGSC) | diaci1.1 |
| *Acyrthosiphon pisum* | i5K pilot project (BCM-HGSC) | Acyr1.0  (Acyr20080623-genome.fa) |
| *Rhodnius prolixus* | Vectorbase | RproC1 |
| *Pediculus humanus* | Vectorbase | PhumU2 |
| *Ladona fulva* | i5K pilot project (BCM-HGSC)/NCBI | GCA_000376725.1 |
| *Ephemera danica* | i5K pilot project (BCM-HGSC)/NCBI | GCA_000507165.1 |
| *Daphnia pulex* | wfleabase | dpulex_jgi060905 |
| *Daphnia pulicaria* | wfleabase | File used: Daphnia_pulicaria_TRO_genome_reads_2004.fasta |
| *Eurytemora affinis* | i5K pilot project (BCM-HGSC)/NCBI | GCA_000591075.1 |
| *Lepeophtherius salmonis* | NCBI Genomes | GCA_000181255.2 |
| *Litopenaeus vannamei* | Penaeus Genome Database (PAGE) | EST collection |
| *Strigamia maritima* | EnsemblMetazoa Genomes | Smar1 |
| *Ixodes scapularis* | EnsemblMetazoa Genomes | IscaW1 |
| *Metaseiulus occidentalis* | i5K pilot project (BCM-HGSC)/NCBI | GCA_000255335.1 |
| *Centruroides exilicauda* | i5K pilot project (BCM-HGSC)/NCBI | GCA_000671375.1 |
| *Parasteatoda tepidariorum* | i5K pilot project (BCM-HGSC)/NCBI | GCA_000365465.1 |
| *Caenorhabditis elegans* | EnsemblMetazoa Genomes | WBcel235 |
| *Biomphalaria glabarata* | Vectorbase | BglaB1 |
| *Crassostrea gigas* | EnsemblMetazoa Genomes | GCA_000297895.1 |
| *Capitella teleta* | DOE Joint Genome Institute/ EnsemblMetazoa Genomes | GCA_000328365.1 |
| *Helobdella robusta* | DOE Joint Genome Institute/ EnsemblMetazoa Genomes | GCA_000326865.1 |
| *Mus musculus* | NCBI Genomes | GCA_000001635.5 |
| *Homo sapiens* | NCBI Genomes | NCBI Genomes |
| *Danio rerio* | NCBI Genomes | GCA_000002035.2 |
| *Amphimedon queenslandica* | EnsemblMetazoa Genomes | Aqu1 |
