# Supplementary material for: Transcriptional Control of an Essential Ribozyme in Drosophila Reveals an Ancient Evolutionary Divide in Animals
Source: PLoS Genet. 2015 Jan 8;11(1):e1004893. doi: 10.1371/journal.pgen.1004893 (PMC4287351; doi:10.1371/journal.pgen.1004893)
Supplement: S2 Table — Publicly available RNA-seq and ChIP datasets used. The RNA-seq data were indexed and sorted using SAMtools [55] and visualized using the Integrative Genomics Viewer [54]. RPR is not a polyadenylated transcript, but it is found in RNA-seq analysis at similar levels in both polyA+-selected and total RNA samples (Fig. 1C). ChIP data from the Berkley Drosophila Transcription Network Project (BDTNP) was visualized using the Integrative Genomics Viewer (Fig. 1D). RPKM values were calculated from the RNA-seq data with Cufflinks [60] and used to generate a heat map (S1 Fig.). References: [52], [63], [64], [71]–[73] (DOCX) [file pgen.1004893.s010.docx]

**Table S2. Publicly available RNA-seq and ChIP datasets used.**

| **Organism** | **Dataset** | **PI and (DCC ID)** | **Reference** |
| --- | --- | --- | --- |
| *Drosophila melanogaster* | total-RNA:Developmental-Stage=Adult-Male,-eclosion-+-5-days#Strain=Y-cn-bw-sp:RNA-tiling-array:Rep-1::Dmel_r5.32 | Celniker, S. (756) | 52 |
| *Drosophila melanogaster* | total-RNA:Developmental-Stage=Adult-Female,-eclosion-+-5-days#Strain=Y-cn-bw-sp:RNA-tilingarray:Rep-1::Dmel_r5.32 | Celniker, S. (755) |  |
| *Drosophila melanogaster* | total-RNA:Developmental-Stage=Embryos-0-2-hr#Strain=Y-cn-bw-sp:RNA-tiling-array:Rep-  1::Dmel_r5.32 | Celniker, S. (101) |  |
| *Drosophila melanogaster* | total-RNA:Developmental-Stage=Embryos-2-4-hr#Strain=Y-cn-bw-sp:RNA-tiling-array:Rep-  1::Dmel_r5.32 | Celniker, S. (118) |  |
| *Drosophila melanogaster* | total-RNA:Developmental-Stage=Embryos-4-6-hr#Strain=Y-cn-bw-sp:RNA-tiling-array:Rep-  1::Dmel_r5.32 | Celniker, S. (120) |  |
| *Drosophila melanogaster* | total-RNA:Developmental-Stage=Embryos-6-8-hr#Strain=Y-cn-bw-sp:RNA-tiling-array:Rep-  1::Dmel_r5.32 | Celniker, S. (122) |  |
| *Drosophila melanogaster* | total-RNA:Developmental-Stage=Embryos-8-10-hr#Strain=Y-cn-bw-sp:RNA-tiling-array:Rep-  1::Dmel_r5.32 | Celniker, S. (103) |  |
| *Drosophila melanogaster* | total-RNA:Developmental-Stage=Embryos-10-12-hr#Strain=Y-cn-bw-sp:RNA-tiling-array:Rep-  1::Dmel_r5.32: | Celniker, S. (105) |  |
| *Drosophila melanogaster* | total-RNA:Developmental-Stage=Embryos-12-14-hr#Strain=Y-cn-bw-sp:RNA-tiling-array:Rep-  1::Dmel_r5.32 | Celniker, S. (107) |  |
| *Drosophila melanogaster* | total-RNA:Developmental-Stage=Embryos-14-16-hr#Strain=Y-cn-bw-sp:RNA-tiling-array:Rep-  1::Dmel_r5.32 | Celniker, S. (109) |  |
| *Drosophila melanogaster* | total-RNA:Developmental-Stage=Embryos-16-18-hr#Strain=Y-cn-bw-sp:RNA-tiling-array:Rep-  1::Dmel_r5.32 | Celniker, S. (111) |  |
| *Drosophila melanogaster* | total-RNA:Developmental-Stage=Embryos-18-20-hr#Strain=Y-cn-bw-sp:RNA-tiling-array:Rep-  1::Dmel_r5.32 | Celniker, S. (113) |  |
| *Drosophila melanogaster* | total-RNA:Developmental-Stage=Embryos-20-22-hr#Strain=Y-cn-bw-sp:RNA-tiling-array:Rep-  1::Dmel_r5.32 | Celniker, S. (115) |  |
| *Drosophila melanogaster* | total-RNA:Developmental-Stage=Embryos-22-24-hrSC#Strain=Y-cn-bw-sp:RNA-tiling-array:Rep-  1::Dmel_r5.32 | Celniker, S. (117) |  |
| *Drosophila melanogaster* | total-RNA:Developmental-Stage=Larvae-L1-stage#Strain=Y-cn-bw-sp:RNA-tiling-array:Rep-1::Dmel_r5.32 | Celniker, S. (93) |  |
| *Drosophila melanogaster* | total-RNA:Developmental-Stage=Larvae-L2-stage#Strain=Y-cn-bw-sp:RNA-tiling-array:Rep-1::Dmel_r5.32 | Celniker, S. (543) |  |
| *Drosophila melanogaster* | total-RNA:Developmental-Stage=L3-stage--12-hr-post-molt-stage-larvae#Strain=Y-cn-bw-sp:RNA-tilingarray: Rep-1::Dmel_r5.32 | Celniker, S. (538) |  |
| *Drosophila melanogaster* | total-RNA:Developmental-Stage=White-prepupae-(WPP)-+-4-days#Strain=Y-cn-bw-sp:RNA-tilingarray: Rep-1::Dmel_r5.32 | Celniker, S. (94) |  |
| *Drosophila melanogaster* | total-RNA:Developmental-Stage=Adult-Female,-eclosion-+-4-days#Strain=Oregon-R#Tissue=Adult-ovaries:RNA-tiling-array:Rep-1::Dmel_r5.32 | Celniker, S. (2340) |  |
| *Drosophila melanogaster* | total-RNA:Developmental-Stage=Adult-Female,-eclosion-+-1-days#Strain=Oregon-R#Tissue=Female-heads:RNA-tiling-array:Rep-1::Dmel_r5.32 | Celniker, S. (2341) |  |
| *Drosophila pseudoobscura* | Poly-A+-RNA;D.pseudoobscura wild-type;Adult Female Whole species; RNA-seq | Oliver, B. (3621) |  |
| *Drosophila virilis* | Poly-A+;D.virilis wild-type;Adult Female Whole species; RNA-seq | Oliver, B. (3613) |  |
| *Drosophila melanogaster* (tissues and cells lines) | All data were obtained from Fly base |  | 71 |
| *Drosophila melanogaster* | Pol-II ChIP-chip |  | 63 |
| *Drosophila melanogaster* | TF-IIB ChIP-chip |  | 64 |
|  |  |  |  |
| *Tribolium castaneum* | SB six hours naive control, Replicate 1 | SRR1048514 | 72 |
| *Tribolium castaneum* | GSM1322429: wildtype1; Tribolium castaneum; RNA-Seq | SRR1161702 |  |
| *Tribolium castaneum* | Whole body Sample; ena-RUN-ANU-01-08-2012-16:01:46:208-1 | ERR161589 | 73 |
