# Supplementary material for: Transcriptional Control of an Essential Ribozyme in Drosophila Reveals an Ancient Evolutionary Divide in Animals
Source: PLoS Genet. 2015 Jan 8;11(1):e1004893. doi: 10.1371/journal.pgen.1004893 (PMC4287351; doi:10.1371/journal.pgen.1004893)
Supplement: S3 Table — Genomic information for the T. castaneum recipient gene (Mbs/PPP1R12B), RPRs, and orthologs of recipient genes in other species. The gene IDs and the alternate IDs in Tcas3.22 (EMBL) identified by a TBLASTN search [61] are shown. Cufflinks was used to determine the locus and gene span [60]. Reference housekeeping genes are shown in blue. (DOCX) [file pgen.1004893.s011.docx]

**Table S3. Genomic information for the *T. castaneum* recipient gene (*Mbs/PPP1R12B*), *RPRs*, and orthologs of recipient genes in other species.**

| **Annotation ID** | **Alternate Annotation** | **Locus** | **Orthrolog** |
| --- | --- | --- | --- |
| TCOGS2:TC003326 | - | ChLG3:1567764-1568895 | Actin |
| TCOGS2:TC006170 | - | ChLG8:4183073-4184078 | Gapdh1 |
| TCOGS2:TC009606 | - | ChLG7:4619508-4621829 | Oda |
| TCOGS2:TC007795 | GLEAN_07795 | ChLG4:907628-908157 | RpL36A |
| TCOGS2:TC011031 | - | ChLG10:10235231-10240344 | eIF-4B |
| TCOGS2:TC003862 | - | ChLG3:26828008-26828844 | Rcc1 |
| TCOGS2:TC003209 | - | ChLG3:149535-163156 | Mbs |
| TCOGS2:TC011455 | - | ChLG10:10262094-10265683 | ATPsynC/CG1746 |
| TCOGS2:TC012619 | - | ChLG9:16626882-16627394 | CG14057/Pop5 |
| TCOGS2:TC002490 | - | ChLG3:32426361-32430029 | Uch (like) |
| CUFF.5683 | - | ChLG5:10171972-10172917 | Rpp30 |
| CUFF.1978 | - | ChLG3:160106-160735 | RNase P RNA |
